# Exosome-mimetic nanoplatforms for targeted cancer drug delivery

**DOI:** 10.1186/s12951-019-0517-8

**Published:** 2019-07-18

**Authors:** Abi J. Vázquez-Ríos, Ángela Molina-Crespo, Belén L. Bouzo, Rafael López-López, Gema Moreno-Bueno, María de la Fuente

**Affiliations:** 1 Nano-Oncology Unit, Translational Medical Oncology Group, Health Research Institute of Santiago de Compostela (IDIS), SERGAS, Choupana Street s/n, 15706 Santiago de Compostela, Spain; 2 University of Santiago de Compostela (USC), Santiago de Compostela, Spain; 3 Translational Cancer Research Laboratory, Department of Biochemistry, Autonomous University of Madrid, School of Medicine, “Alberto Sols” Biomedical Research Institute CSIC-UAM, IdiPaz, Madrid, Spain; 4 MD Anderson International Foundation, 28033 Madrid, Spain; 5 Cancer Network Research (CIBERONC), 28029 Madrid, Spain

**Keywords:** Exosomes, Exosome-mimetic nanoplatforms, Biomimetics, Drug delivery systems, Gene therapy, Cancer

## Abstract

**Background:**

Lack of effective tumor-specific delivery systems remains an unmet clinical challenge for successful translation of innovative therapies, such as, therapeutic oligonucleotides. In the past decade, exosomes have been suggested to be ideal drug delivery systems with application in a broad range of pathologies including cancer, due to their organotropic properties. Tumor-derived exosomes, having tumor-homing properties, can efficiently reach cancer cells and therefore behave as carriers for improved drug delivery to the primary tumor and metastases. However, due to their complex composition, and still undefined biological functions, safety concerns arise hampering their translation to the clinics.

**Results:**

We propose here the development of exosome-mimetic nanosystems (EMNs) that simulate natural tumor-derived exosomes with respect to their structure and functionality, but with a controlled composition, for the targeted delivery of therapeutic oligonucleotides to lung adenocarcinoma cells (microRNA-145 mimics). Making use of the well-known liposome technology, EMNs can be engineered, loaded with the therapeutic compounds, and tailored with specific proteins (integrin α6β4) providing them organotropic properties. EMNs show great similarities to natural exosomes with respect to their physicochemical properties, drug loading capacity, and ability to interact with the cancer target cells in vitro and in vivo, but are easier to manufacture, can be produced at high yields, and are safer by definition.

**Conclusions:**

We have designed a multifunctional nanoplatform mimicking exosomes, EMNs, and proved their potential to reach cancer cells with a similar efficient that tumor-derived exosomes but providing important advantages in terms of production methodology and regulations. Additionally, EMNs are highly versatile systems that can be tunable for a broader range of applications.

**Electronic supplementary material:**

The online version of this article (10.1186/s12951-019-0517-8) contains supplementary material, which is available to authorized users.

## Background

Recent data regarding the outcome of cancer highlight the need for more effective and innovative therapies. It is generally accepted that selectively reaching tumor cells will allow for more effective treatments without toxic side effects [1]. Given their role in intercellular communication, exosomes fulfill the requirements of an ideal drug delivery system. They can (i) transport molecules, (ii) cross biological membranes, (iii) overcome peripheral macrophages, and (iv) reach specific cell types to release their content [2]. Exosomes loaded with anticancer drugs have already shown promise as a new therapeutic approach in animal models [3–7]. Using tumor-derived exosomes will provide additional competitive advantages for the selective delivery of anticancer therapies not only to the primary tumor but also to metastasis and even to the premetastatic niche, owing to their intrinsic organotropic tumor-homing properties [8, 9]. Besides, tumor-derived exosomes are also involved in a wide range of biological processes, including tumor progression, metastasis formation, and drug resistance, mechanisms that still remain poorly understood, making it necessary to generate a deeper knowledge before considering their safe application in therapeutics [10, 11].

Referring to the application of exosomes as cancer drug delivery systems, and besides the increasing interest overseen over the last years, only plant-derived exosomes loaded with curcumin for the treatment of colon cancer are currently under clinical evaluation with an ongoing phase I clinical trial (NCT01294072). This low translation rate might be related to the tedious and time-consuming isolation processes, as well to the lack of standardization regarding the production, characterization, and quality control assessment of the isolated exosomes. Safety concerns, due to their still unknown compositions and undescribed functionalities, severely hamper the clinical application of tumor-derived exosomes.

The potential of nanotechnology for developing improved anticancer therapies can be summarized in an increasing number of nanomedicines approved for the treatment of cancer [12]. However, the concept of the ‘magic bullet’, popularized by Paul Ehrlich at the beginning of the XX century, is still an ambitious objective in the field of nanotechnology, drug delivery, and cancer [1]. Nanostructures that resemble tumor-derived exosomes, both structurally and functionally, can provide a real alternative for the development of targeted anticancer therapies. It is generally accepted that exosomes are enriched in certain lipid species and share common groups of proteins, some of them responsible for their tumor-homing properties [9, 13, 14]. Therefore, by the combination of these materials and selection of an appropriate production methodology, it is possible to engineer EMNs.

We hence propose the development of EMNs, by integrating key components of tumor-derived exosomes, and provide evidence of their potential for the targeted delivery of anticancer therapeutics to the target site, while overcoming the main limitations of their natural counterparts.

## Results

### Development and characterization of a nanoplatform structurally similar to exosomes

Our purpose was to design a nanoplatform that resemble exosomes for their composition and physicochemical properties, of utility for the selective delivery of anticancer therapies. For reference, we isolated and characterized cell-derived extracellular vesicles (Additional file 1: Figure S1). WB analysis allowed confirming the effectiveness of the isolation protocol (Additional file 1: Figure S1a), and extensive proteomic analysis (LC–MS/MS) led to the identification of other 90 exosomal protein markers described in the top 100 proteins of the ExoCarta and EV Vesiclepedia databases (Additional file 1: Figure S1b). Nearly 80% of the identified proteins were also classified as part of “exosome component” in the Gene Ontology Cellular Component section of the FunRich tool (Additional file 1: Figure S1c). Exosomes with characteristic cup-shape morphologies were observed by TEM (Additional file 1: Figure S1d), their yield of production determined (Additional file 1: Figure S1e) and their physicochemical properties measured by DLS and LDA (Table 1).

**Table 1:** Physicochemical properties of exosomes and EMNs

	Size (nm)^a^	PdI	ZP (mV)
Exosomes from human plasma	98 ± 12	0.4	− 16 ± 1
Exosomes from cancer cell lines	91 ± 11	0.3	− 23 ± 2
EMNs	100 ± 8	0.2	− 7 ± 2
F-EMNs	110 ± 2	0.3	− 6 ± 1
EMNs + miR145	104 ± 2	0.3	− 16 ± 2
F-EMNs + miR145	113 ± 1	0.3	− 5 ± 2

Data presented as mean ± standard deviation; n = 3

*PdI* polydispersity index, *ZP* zeta potential, *EMNs* Exosome-mimetic nanosystems, *F-EMNs* functionalized EMNs with a specific integrin, *miR145* microRNA-145, *EMNs + miR145* EMNs loaded with miR145, *F-EMNs + miR145* F-EMNs loaded with miR145

^a^Size corresponds to number measurement in DLS

**Fig. 1:**
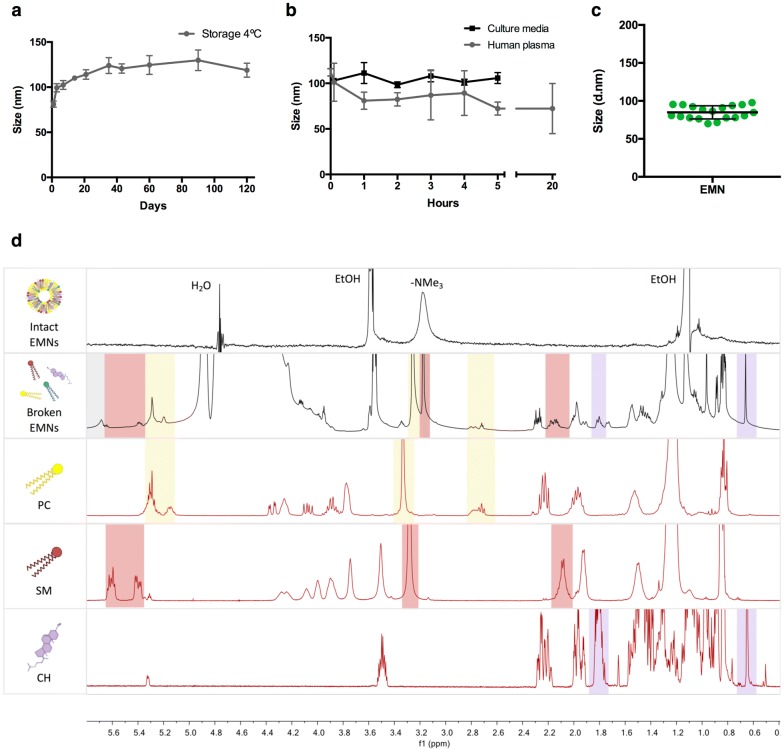
Exosome-mimetic nanoplatforms characterization. **a** Hydrodynamic size (Z-average) of EMNs measured by dynamic light scattering (DLS) under storage conditions (4 °C) and **b** in culture media and human plasma, over the time (from t = 0 to 120 days, 5 h and 20 h, respectively; n = 6). **c** Hydrodynamic size measured by DLS of 19 independent batches of EMNs (green dots); horizontal bar represents mean ± SD. **d** ^1^H-NMR spectrum showing representative signals of each component found in intact (D_2_O) and broken EMNs (MeOD) compared to the spectrum of the pure components (PC, SM, and CH). Specific peaks of each component identified in the sample of broken EMNs were highlighted with colored rectangles (PC, yellow rectangles; SM, red rectangles; CH, purple rectangles)

We next attempted the preparation of EMNs. We took into consideration previously published lipidomics works describing the most common lipid species enriched in tumor exosomes compared to parent cells, to define their composition (Additional file 1: Figure S2a) [13–15]. We optimized the preparation methodology based on previous knowledge using the ethanol injection method (we studied the influence of several parameters, lipid concentration, volumes, and the ratio of the components, in the physicochemical properties of the resulting liposomes), in order to obtain nanoplatforms structurally similar to exosomes in a single step (Additional file 1: Figure S2b). The properties of the obtained EMNs (schematically represented in Additional file 1: Figure S2c) are disclosed in Table 1. EMNs composition can be further modulated in order to obtain nanovesicles of different sizes and properties (Additional file 1: Table S1). Stability studies revealed great colloidal stability during storage (Fig. 1a), and upon incubation with cell culture media and human plasma (Fig. 1b). Characterization of 19 independent batches confirmed the excellent reproducibility of the formulation **(**Fig. 1c). NMR results (^1^H and ^31^P NMR) prove that each lipid component was effectively incorporated into EMNs (Fig. 1d). While the spectrum corresponding to broken EMNs displayed relevant peaks corresponding to the free lipid molecules (according to the spectra of the pure components PC, SM, and CH, colored rectangles), the intact EMNs spectrum did not display practically any peak (only a terminal methyl group), meaning that free unreacted lipids were not present in the suspension. The ^31^P NMR spectrum was also acquired for a double-checking, allowing to observe two peaks (broken EMNs) corresponding to the phosphorylated species of SM and PC. Moreover, this analysis resulted in a proportion of 0.48:1 (SM:PC), similar to the theoretical proportion 0.44:1, revealing that each lipid was efficiently and completely incorporated in EMNs in the exact proportion used for their preparation (Additional file 1: Figure S3).

**Fig. 2:**
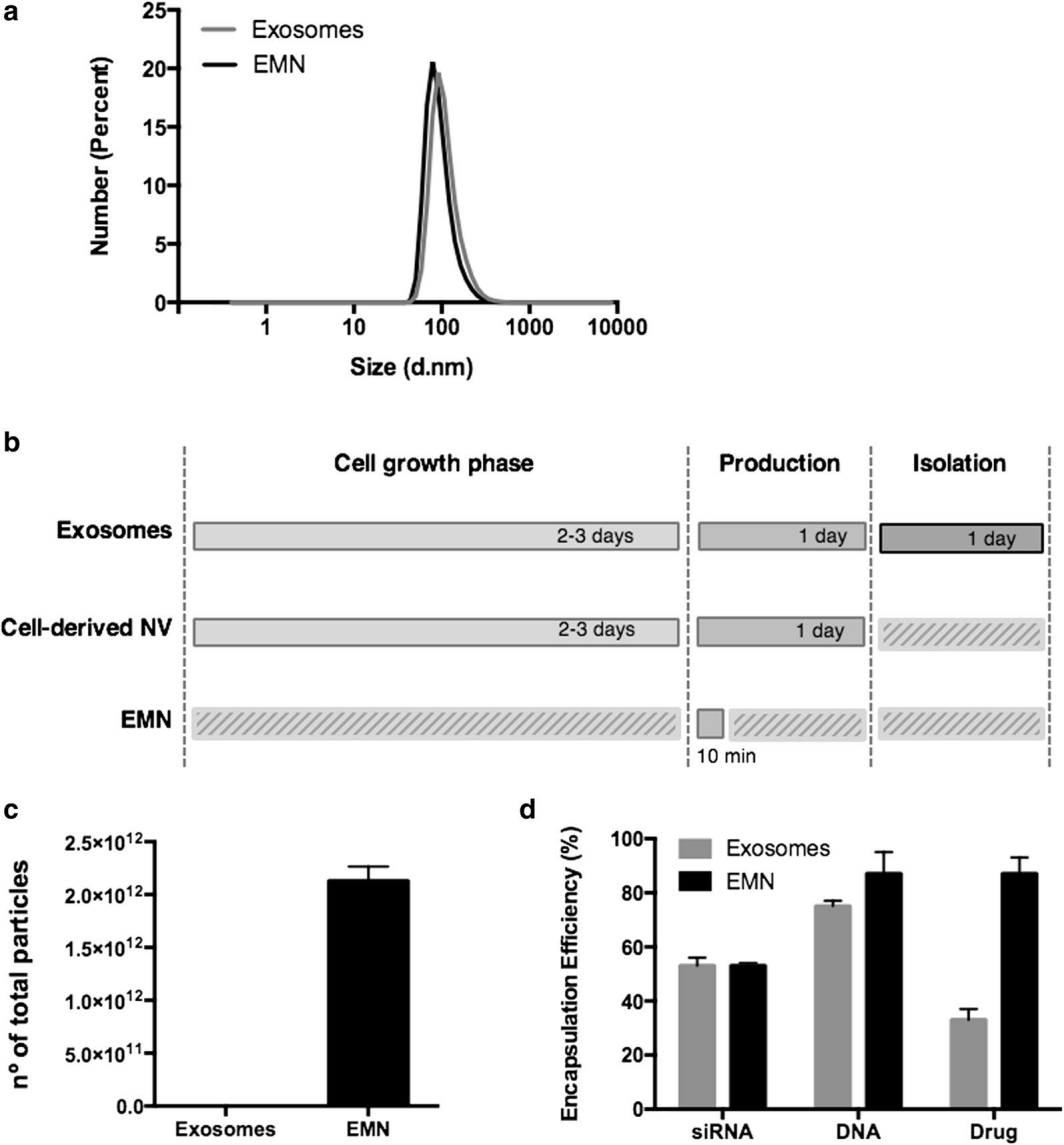
Comparison between natural exosomes and EMNs. **a** Size distribution measured by DLS of natural exosomes and EMNs showing that we have obtained nanoplatforms of practically the same size than natural exosomes. **b** Time-consuming comparison for obtaining natural exosomes from cell lines and isolation by serial ultracentrifugation, cell-derived nanovesicles (NV) and EMNs. **c** The number of particles obtained in 216 ml of conditioned medium (16 h) of A549 exosomes and one batch of EMNs (2′2 ml). **d** Encapsulation efficiencies of therapeutic model molecules comparing the loading capacity of natural exosomes and EMNs. Bar charts represent mean ± standard deviation, n = 3

We provide a methodology for preparation of EMNs structurally similar to exosomes (Table 1, Fig. 2a), while overcoming important limitations: (i) a single batch of EMNs (2′2 ml), can be produced in 10 min, while the exosome isolation time from conditioned medium by serial ultracentrifugation (Additional file 1: Figure S1e, protocol detailed in Additional file 1: Methods) takes several days, therefore offering a time-saving efficiency of at least 5 days for each production run; this production process is also faster than other current alternatives to exosomes, such as cell-derived nanovesicles by serial extrusion, which also requires time for cell growing and production [16] (Fig. 2b), (ii) the production yield, measured by the number of obtained particles, is 1000-fold higher for EMNs than for exosomes (Fig. 2c), (iii) EMNs have a similar drug loading capacity than exosomes in the case of RNA (hydrophilic compound), and superior drug loading efficiencies in the case of DNA modified with a cholesterol chain (amphiphilic compound), and hydrophobic compounds such as curcumin (Fig. 2d), (iv) EMNs can be efficiently internalized by different cancer cells, and subsequently, deliver their payload intracellularly (Additional file 1: Figure S2d) without showing toxicity (Additional file 1: Figure S2e), (v) lipids are cheap and well-characterized materials, and finally and very importantly from a translational perspective, (vi) industrial production processes under GMP conditions are already well established for liposomes [17].

### Association of bioactive macromolecules

**Fig. 3:**
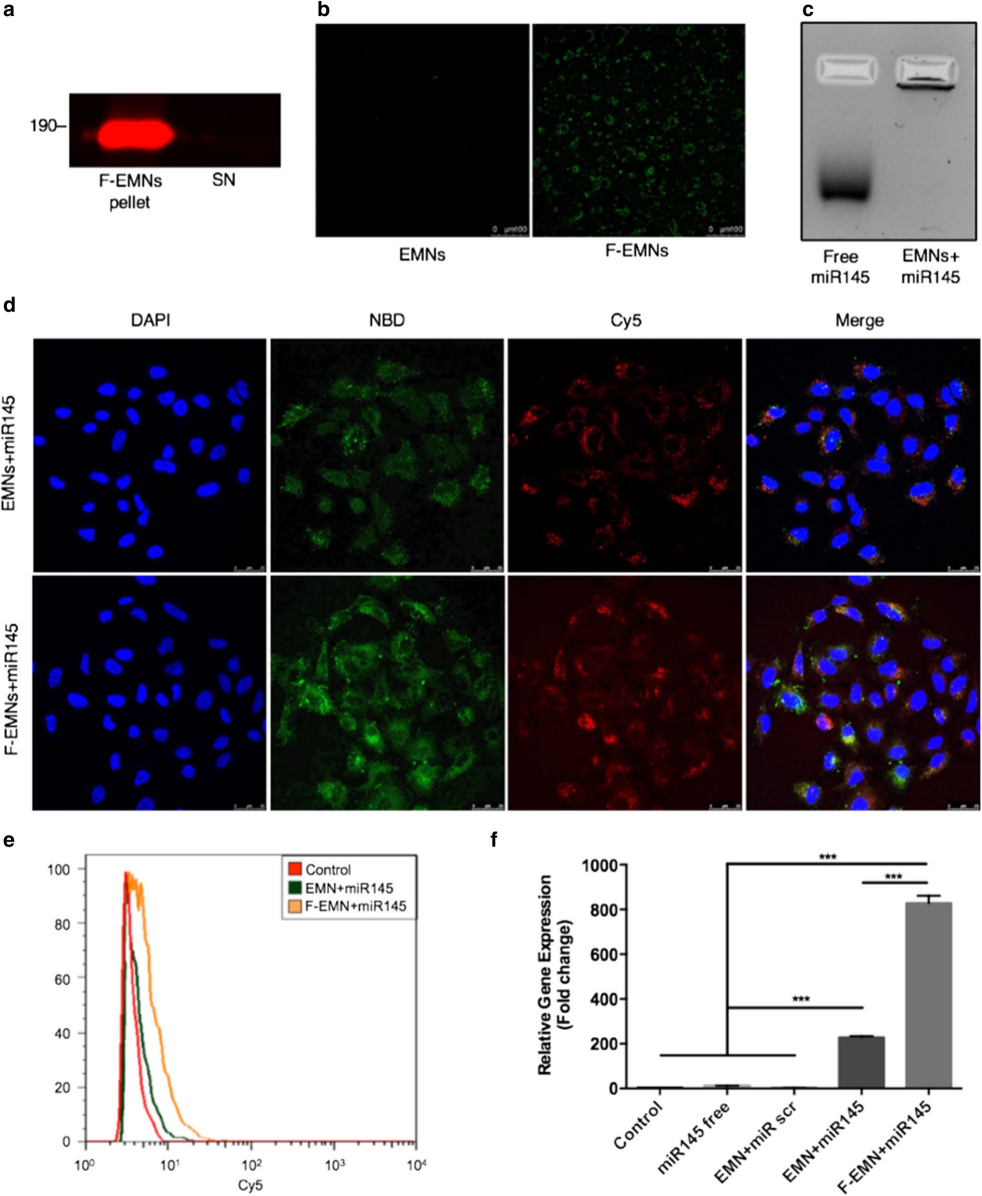
Functionalization of EMNs with ITGα6β4 (F-EMNs) and delivery of miRNA145. **a** Fluorescent Western blot showing the effective association of ITGα6β4 with EMNs (F-EMNs) by ultracentrifugation at 120,000×*g* (pellet) compared to the supernatant (SN). **b** Specific interaction of F-EMNs to coverslips coated with laminin-5. EMNs and F-EMNs were labeled with the fluorophore NBD-CH (green). Scale bars represent 100 µm. **c** Gel retention assay showing the free microRNA145 compared to the miR145 encapsulated in EMNs that remains stacked in the well of the gel. **d** Confocal microscopy images after 4 h transfection of A549 cells with EMNs + miR145 and F-EMNs + miR145. Blue channel: nuclei (DAPI); green channel: EMNs and F-EMNs (NBD-CH); red channel: miR145 (Cy5). Scale bars represent 25 µm. **e** FACS quantitative analysis of the transfection efficiency of F-EMNs + miR145-Cy5 compared to EMNs + miR145-Cy5, and the control cells. **f** Real time-qPCR of miR145 levels in A549 cells after 4 h transfection with miR145 free, a scramble sequence, EMNs + miR145 and F-EMNs + miR145. Data are representative of three independent experiments

We next increased the complexity of the formulation to include bioactive macromolecules, RNAs and proteins, to obtain EMNs with similar functionalities to simplified exosomes (Table 1). EMNs showed a good capacity to associate different types of proteins, irrespective of their MW and pI, without observation of significant changes in their physicochemical properties (nanoparticle size, distribution, and surface charge) and with association efficiencies over 80% (Additional file 1: Table S2, Additional file 1: Figure S4). This is particularly important, allowing envisioning a versatile nanosystem that can be tailored with active proteins providing organotropic functionalities. Exosomal ITGα6β4, related with lung organotropism [9], was successfully bound to EMNs as verified by fluorescent WB (Fig. 3a shows a red signal in the loading well that corresponds to the protein bound to the EMNs), with the aim of increasing their targeting capability to the lung and enhancing their adhesive properties to recipient cells. The resulting EMNs functionalized with ITGα6β (F-EMNs), and labeled with NBD, had the capacity to mediate specific and effective interactions with laminin-5, according to a binding assay performed with laminin-5 coated plates. NBD-labeled plain EMNs were included in the experiment as the control formulation (Fig. 3b). Additionally, F-EMNs were perfused through a 3D culture model, previously developed by our group [18], in order to evaluate their interaction with A549 cells that express laminin-5 under dynamic conditions (Additional file 1: Figure S5a). Confocal images confirmed an efficient interaction of F-EMNs with the cells and show a good colocalization with laminin-5 (Additional file 1: Figure S5b).

Subsequent experiments were conducted to explore the potential of F-EMNs to transport RNAs to cancer cells. Besides cationic lipids are not present in the composition [19], we proved that EMNs efficiently associate therapeutic oncosuppressor miR145 by performing an agarose gel retention assay (Fig. 3c). Hence, we eventually engineered EMNs that simultaneously incorporate lipids, proteins, and RNAs, and resemble simplified exosomes with respect to their composition, physicochemical properties, and functionalities (Table 1). Confocal images proved that EMNs mediate an efficient delivery of miR145-Cy5 to A549 cells and support our notion that integrin functionalization indeed increases the adhesive properties in the case of F-EMNs as well as the transfection efficiency (Fig. 3d, e). Moreover, looking at the 3D reconstruction of the confocal images (Additional file 1: Figure S4c) and xz and yz-slices (Additional file 1: Figure S4d), a clear colocalization of NBD-labeled F-EMNs and miR145-Cy5 was observed, proving that the payload and the carrier are traveling together. Also, EMNs provide great opportunities for being further amendable to add different fluorophores for broader applications (Additional file 1: Figure S6 depicts triple labeling of EMNs with TopFluor-SM, Cy5 and DiR**)**. RT-PCR assays confirmed the superior behavior of F-EMNs for delivery of therapeutic RNAs to cancer cells, which rendered a fivefold increase in the expression of miR145 with respect to the control formulation without ITGα6β4 (EMNs + miR145) (Fig. 3f). As expected, control cells transfected with miR145 in solution (miR145 free) or with EMNs loaded with a scrambled sequence (EMNs + miRscr) did not show differences in the expression of miR145 compared to untreated cells. This increase in the intracellular levels of miR145 produced by F-EMNs was translated into a significant reduction of the clonogenic capacity of the transfected cells (Additional file 1: Figure S7a). Additionally, to evaluate whether F-EMNs could be trapped in the endolysosomal degradation pathway [20], a lysosomotropic agent that induces lysosomal membrane perturbation, called chloroquine [21, 22], was added to the cells previously transfected. Results of a colony formation assay showed that no significant differences were observed when chloroquine was added, proving that F-EMNs can efficiently escape the endolysosomal system (Additional file 1: Figure S7b). Moreover, at the protein level, F-EMNs loaded with miR145 significantly reduced the expression of N-cadherin in the transfected cells, a protein that promotes tumor cell survival, migration, and invasion, and one of the targets of miR145 in lung adenocarcinoma [23] (Additional file 1: Figure S7c). Therefore, F-EMNs are able to deliver functional miR145 to cancer cells, overcoming the main barriers in gene delivery.

### F-EMNs have a similar capacity than tumor-derived exosomes to transport therapeutic RNAs to cancer cells

**Fig. 4:**
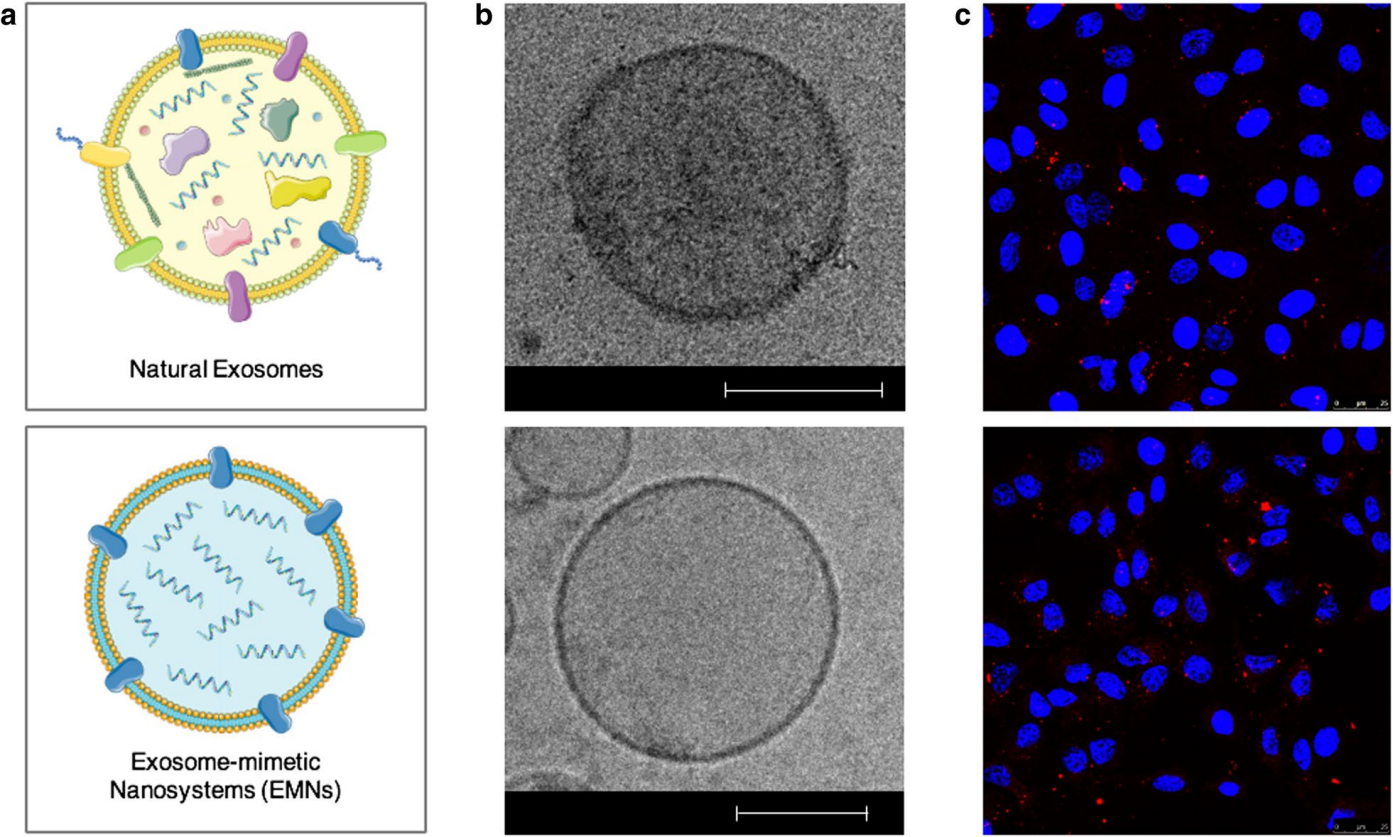
Comparison of natural exosomes and EMNs morphology and miR145 association. **a** Schematic representation of the composition and morphology of natural exosomes (upper) and functionalized miR145-loaded EMNs (lower). **b** Cryo-TEM images of natural exosomes loaded with miR145 (upper) and F-EMNs + miR145 (lower). Scale bar represents 100 nm. **c** Confocal images of miR145 delivery by natural exosomes (upper) and F-EMNs (lower) in A549 cells. Blue channel: nuclei (Hoechst); red channel: microRNA145 (Cy5). Scale bars represent 25 µm

**Fig. 5:**
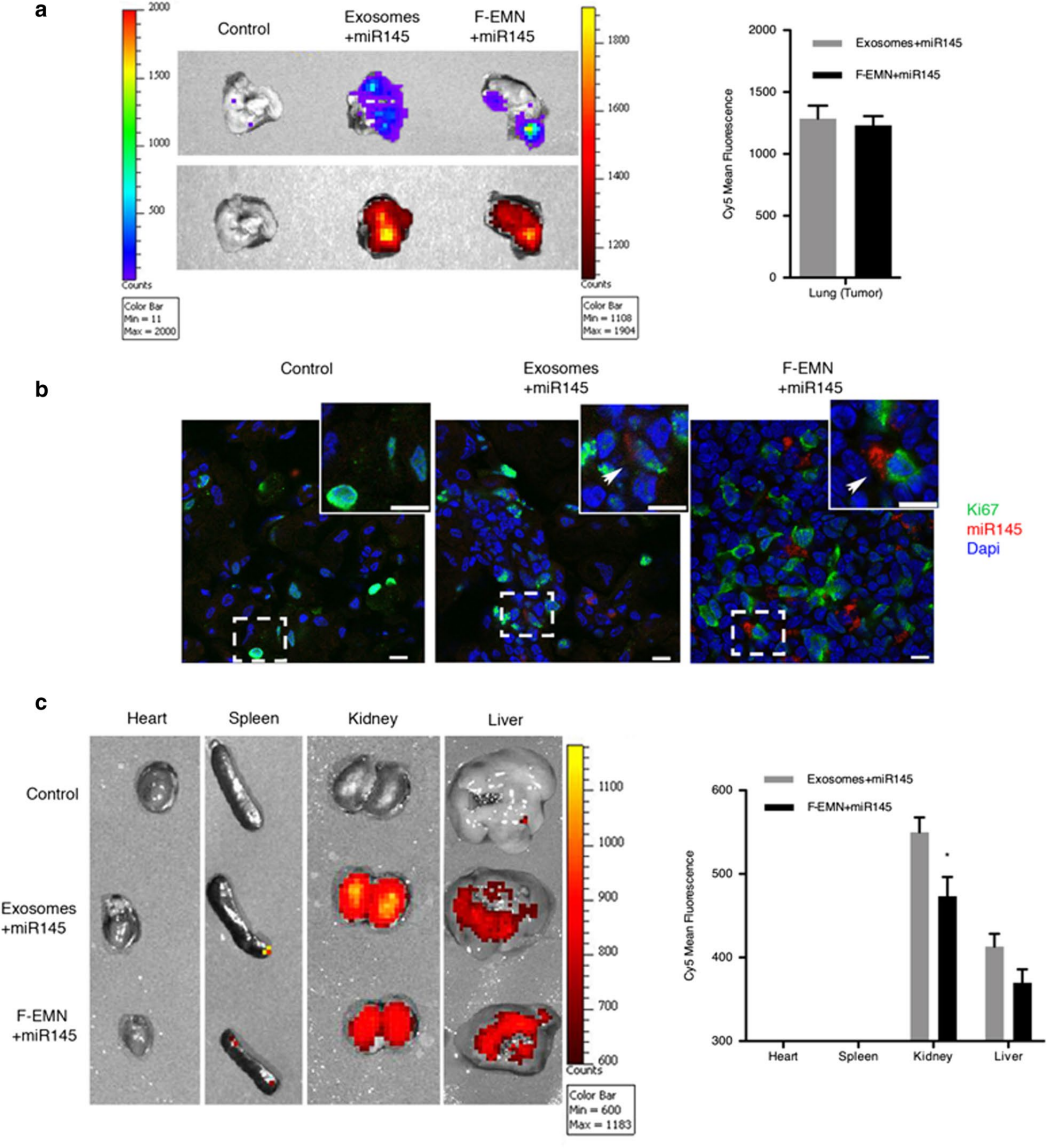
In vivo biodistribution of natural exosomes and F-EMNs loaded with miR145-Cy5. **a** Representative image ex vivo (left) and quantification of the Cy5 emission (right) of the lung (tumor) after treatment with natural exosomes or F-EMNs. The luminescence signal (up) and Cy5 fluorescence (down) of the same tumor were shown. **b** Representative confocal microscopy images of lung tumor cryosections stained with DAPI (blue) and ki67 (green) for analyzing the miR145-Cy5 intracellular uptake in vivo (red) in the metastatic cells after the same treatments indicated in (a). The arrows label the areas of miR145-Cy5 accumulation and the scale bar represents 10 μm. **c** Representative image ex vivo (left) and quantification of the Cy5 emission (right) of the indicated organs. The scale bars (**a**, **c**) represent the luciferase intensity (left) and Cy5-fluorescence (right, arbitrary units). The data in graphs (**a**–**c**) denote the mean values ± SEM from n = 5 mice per condition and Cy5 fluorescence signal was normalized to the background obtained from tumors of mice control. *p < 0.05. Data without statistical significance were not indicated

Final experiments were established to compare tumor-derived exosomes with engineered F-EMNs loaded with miR145, both represented in Fig. 4a. Cryo-TEM images revealed similarities with respect to the vesicle size, spherical shape, and membrane thickness (Fig. 4b). Transfection experiments in vitro proved that miR145-loaded F-EMNs are able to transport genetic material to tumor A549 cells in a comparable fashion to exosomes isolated from the same cell culture, as it can be seen by confocal microscopy pictures and FACS analysis (Fig. 4c, Additional file 1: Figure S8). Next, in vivo biodistribution experiments in mice bearing lung cancers (inoculation of luciferase-expressing A549 lung carcinoma cells into the tail vein of nude mice leading to tumor formation) show a comparable behavior of F-EMNs and tumor-derived exosomes both loaded with miR145-Cy5. First experiments were conducted to determine the most suitable administration route. miR145-Cy5-loaded F-EMNs were administered either intraperitoneally (IP) or retro-orbitally (RO), since by these routes, lungs are not reached directly after injection. Results prove that F-EMNs + miR145-Cy5 could efficiently reach the tumors irrespective of the administration route (Additional file 1: Figure S9a), but a higher fluorescent signal in the liver, kidney, and spleen, was observed after RO in comparison to IP injection, indicative of a higher accumulation in these organs following the first administration route (Additional file 1: Figure S9b). Importantly, we did not observe any signal in the heart, supporting the idea that F-EMNs treatment would avoid cardiotoxicity, one of the main concerns related to the development of cancer therapeutics [24]. According to these results, we decided to pursue with the IP modality, which also proved to be adequate for administration of other types of nanocarriers loaded with biomolecules [25]. We injected miR145-Cy5-loaded F-EMNs and miR145-Cy5-loaded tumor-derived exosomes and confirmed that both treatments could mediate the accumulation of miRNA in the lung and the tumor with similar efficiency (Fig. 5a). Immunofluorescent studies with the excised tissue revealed localization of miR145-Cy5 in the neighborhood of Ki67 positive proliferative tumor cells (Fig. 5b). In addition, there was a significant reduction in the fluorescence signal observed in the kidney and the liver for mice receiving F-EMNs + miR145-Cy5 with respect to mice receiving exosomes, suggesting that F-EMNs can reach to the tumor efficiently, and probably provide lower systemic toxicities (Fig. 5c).

## Discussion

Given their role in intercellular communication, exosomes are being increasingly explored as delivery systems for biomedical purposes. The organotropism described for tumor-derived exosomes to the tumor site and premetastatic niche could hold the key for designing highly efficient anticancer therapies targeting metastatic cancer with minimum side effects [9, 26]. However, the main drawback for the application of this approach is the lack of more in-depth understanding of their molecular composition and function, raising safety concerns that must be resolved prior to promote their therapeutic application [10]. Moreover, the low yield, highly laborious, costly, and time-consuming methods of production for cell-derived exosomes, together with a lack of standardization for relevant processes such are determining their physicochemical properties and drug loading capacity, are additional challenges to overcome [27–29]. Researchers have tried to overcome some of these challenges by pursuing different strategies such as the generation of nanovesicles by serial cell extrusion, or the use of cell membranes (e.g. from erythrocytes, platelets, mesenchymal cells-MSC) for the coating of polymeric nanoparticles, rendering what they named exosome-mimics or synthetic exosomes [30–34]. However, these approaches do not overcome the challenges related to off-target signaling from proteins present in the vesicle surface, or the undesirable delivery of additional species present in the lumen [29].

In an attempt to overcome technical limitations and regulatory issues related to the clinical use of exosomes in cancer drug delivery, we have pursued a rational design of EMNs based on the well-known liposome technology. Liposomes are the most widely studied types of nanosystems and have successfully been translated into clinical products [17]. Therefore, EMNs can benefit for the accumulated knowledge in industrial scaling-up and GMP production of liposomes. Moreover, considering that liposomes are typically used as controls for preclinical evaluation of exosomes as drug delivery carriers, rational-designed EMNs can also have an application in this regard. Indeed, a recent review by Johnsen et al. highlights the crucial need for an adequate choice of liposomal controls in preclinical exosome-based drug delivery studies before postulate any potential superiority of exosomes over their liposomal counterparts [35]. Authors often choose over-simplified liposomes for their comparisons and rarely would choose a clinically relevant liposomal formulation. Gold standard liposome controls are urgently needed for a much fairer comparison.

EMNs provide additional opportunities of being further amenable to surface functionalization as well as loading of therapeutic cargo for a broader application in other fields. From our perspective, rather than just for competitive purposes, our nanoplatform could also serve as a tool for a deeper understanding of the still not answer questions of exosomes, for instance, what is the role of specific lipid species or proteins in the membrane surface of exosomes in trafficking, cellular uptake, or cell-to-cell interaction processes. The answers to these questions would benefit both fields for reaching sooner a novel clinically relevant drug delivery system.

Taking into account all these considerations, we have successfully engineered EMNs, mimicking simplified natural exosomes not only structurally, but also holding specific molecular features and functionalities (F-EMNs + miR145). EMNs are prepared in very mild conditions (e.g. avoiding pH changes and high-energy processes), using an optimized methodology that is fast, simple, and reproducible. Using the ethanol injection methodology, we are able to produce small nanoplatforms in few minutes without needing additional steps for adapting the size and lamellarity (e.g. extrusion or sonication) and keeping the amount of ethanol in the formulation below 10%, meaning that it does not need to be removed prior to in vivo administration [36]. This preparation method resulted in a substantially increased production yield and reduced preparation time with respect to isolated tumor-derived exosomes. Moreover, these nanoplatforms are only composed of natural lipids enriched in exosomes [13]. Their use has been widely reported in other clinical formulations, therefore having a well-known safety profile. Importantly, the lipidic composition can be easily tailored, allowing incorporation of additional species that could be described in future for having relevant roles in trafficking, cell communication, or cell-to-cell interaction processes, accompanying advances in the lipidomic field [15, 37]. Lastly, EMNs efficiently incorporate labile macromolecules (i.e. RNA and proteins). Encapsulation of therapeutic proteins into liposomes has been attempted with great success in the case of insulin, calcitonin, VIP, and interleukins [38]. In our hands, we successfully functionalized our nanoplatforms with the extracellular fraction of the transmembrane protein integrin α6β4, that can be found in tumor-derived exosomes conferring tumor homing properties and proved that its presence improves the adhesion properties of EMNs and ability to deliver the associated therapeutics to cancer cells. EMNs can also associate miRNAs with comparable efficiency to exosomes, besides they are neutrally charged. Typically, nanocarriers for gene delivery incorporate cationic compounds bearing intrinsic toxicity [39], but neutral ones have recently been claimed a much safer alternative avoiding off-target effects [40]. Transfection studies show that our nanoplatforms functionalized with integrins, F-EMNs, can mediate an increase in the intracellular levels of miR145 of over 800-fold higher than the free miR145, and fivefold higher than the control formulation (EMNs). This increased expression related to relevant changes in cancer cells phenotype. In vivo experiments carried out in a lung cancer mice model allowed us confirming that F-EMNs show a similar capacity to transport their therapeutic cargo (miR145) to the target as compared to tumor-derived exosomes. Importantly, F-EMNs do not apparently provide cardiotoxicity and show a lower accumulation by the liver and kidney than their natural counterparts.

## Conclusions

Overall, we have designed a multifunctional nanoplatform mimicking exosomes, F-EMNs loaded with RNAs, that can be manufactured and characterized in a controlled manner for a safer biomedical approach, and have demonstrated to efficiently transport bioactive macromolecules to the target cells in a similar fashion to tumor-derived exosomes. We provide the first proof-of-concept of the potential of this technological nanoplatform as a real alternative to exosomes for the development of safer and more efficient anticancer therapies, a technology that is versatile and can be adapted as we go deep in the study of exosomes and the molecular features related to their tumor-homing properties.

## Methods

### Materials

Phosphatidylcholine (Lipoid E PC) and sphingomyelin (Lipoid E SM) were obtained from Lipoid GmbH (Ludwigshafen, Germany). Cholesterol was purchased from Sigma-Aldrich (Madrid, Spain). C16 Ceramide, NDB-6 Cholesterol, and C11 TopFluor Sphingomyelin, were all purchased in Avanti Polar Lipids (Alabaster, AL, USA). MilliQ^®^ water (Simplicity 185, Millipore, Bedford, USA) was used throughout the study. Ethanol of analytical grade was purchased from VWR (Barcelona, Spain). DiR (DiIC18(7) (1,1′-Dioctadecyl-3,3,3′,3′-Tetramethylindotricarbocyanine Iodide)) and DiD (1,1′-dioctadecyl-3,3,3′,3′-tetramethylindotricarbocyanine perchlorate) were acquired from Thermo Fisher Scientific (USA). miRNA-145 (miR145; sense strand 5′-GUCCAGUUUUCCCAGGAAUCCCU-3′, antisense strand 5′-GGAUUCCUGGAAAUACUGUUCU-3′), miRNA-145-Cy5 (miR145-Cy5,), miRNA-scramble (miRscr) and a model siRNA were synthesized by Eurofins Genomics (Ebersberg, Germany). DNA-CH was kindly provided from Ramon Eritja (Nucleic Acids Chemistry Group, Institute for Advanced Chemistry of Catalonia, Barcelona, Spain).

### Cell culture

SW480 (ATCC^®^ CCL-228), PC-3 (ATCC^®^ CRL-1435) and A549 (ATCC^®^ CCL-185) cells were grown in Dulbecco’s modified Eagle’s medium (DMEM) high glucose (Gibco, Thermo Fisher Scientific), supplemented with 10% fetal bovine serum (FBS) (Thermo Scientific, Spain), and 1% penicillin/streptomycin (Thermo Scientific, Spain). Cells were maintained at 37 °C in a 5% CO_2_ humidified atmosphere. Trypsin and Phosphate Buffered Saline (PBS) were purchased from Sigma-Aldrich (St. Louis, USA). All cell lines were tested routinely and confirmed to be mycoplasma-free. The A549 cells were authenticated by STR-profiling according to ATCC guidelines.

### EMNs preparation and characterization

#### Preparation of EMNs

EMNs with a well-defined composition (CH:PC:SM:Cer) were prepared following the ethanol injection methodology. In brief, lipids were dissolved in ethanol, at defined ratios (0.9:1:0.4:0.03 w/w), and a total lipid concentration of 0.92 mg/ml. 200 μl of the ethanol solution was injected with an insulin syringe (0.5 ml, 0.33 × 12 mm ICO.C.1) into 2 ml of milliQ water, under magnetic stirring. EMNs were spontaneously formed.

#### Dynamic light scattering (DLS) and laser Doppler anemometry (LDA)

The hydrodynamic diameter, polydispersity index and superficial charge of the exosomes and EMNs were measured using a Zetasizer Nano ZS (Malvern Instruments, UK). Measurements were performed in PBS 1X (exosomes) and MilliQ water (EMNs) at room temperature (RT). For the zeta potential measurements, samples were diluted in 1 mM potassium chloride (KCl).

#### Nanoparticle tracking analysis (NTA)

Particle size and concentration distribution of the EMNs and exosomes were also measured using NTA (v2.3; Malvern Instruments, Malvern, UK) according to manufacturer’s instructions. Briefly, EMNs samples were vortexed and diluted to a final dilution of 1:1000 in milliQ H2O and exosomes 1:100. Blank-filtered H_2_O was run as a negative control. Each sample analysis was conducted for 60 s and measured five times using Nanosight automatic analysis settings. The detection threshold was set to level 11 and camera level to 15.

#### Stability

A stability study was performed in human plasma and cell culture media (DMEM supplemented with 1% FBS) incubated at 37 °C. The colloidal properties of EMNs were determined using the Zetasizer Nano ZS each hour up to 5 h for human plasma and 20 h for DMEM. Stability of the formulation under storage conditions was also tested in PBS 1X at 4 °C up to 3 months. All measurements were performed in sextuples.

#### Nuclear magnetic resonance spectroscopy (NMR)

NMR experiments relying on ^1^H and ^31^P detection were measured on a Varian Inova 17.6 T spectrometer (750 MHz proton resonance) equipped with a triple resonance HCP probe and z-gradient. The ^13^C NMR spectra were measured on a Varian Mercury 7.04 T (75.4 MHz, ^13^C resonance) equipped with a double resonance ATB probe with z-gradient. The spectra were processed and analyzed with MestreNova software v11.0 (*Mestrelab. inc.*).

#### Cryogenic transmission electron microscopy (cryo-TEM)

Samples were initially vitrified according to Dubochet protocol [41]. Briefly, an aliquot of 3.5 μl of each sample was applied to glow-discharged holey grids for 1 min, blotted, and rapidly plunged into liquid ethane at − 180 °C and kept at this temperature until visualization. Images were obtained at 0°-tilt under minimum dose conditions using a field emission gun Tecnai 20 G2 Microscope (FEI, Eindhoven, The Netherlands) equipped with a Gatan cold stage operated at 200 keV. Low-dose images were collected at a nominal magnification of ~ 50,000× by using an FEI Eagle CCD camera with a step size of 15 μm. The original pixel size of the acquired images was 2.74 Å.

### Loading of therapeutic molecules

#### Exosomes

Exosomes were loaded with different molecules. The hydrophobic drug Curcumin (Acros Organics™, Thermo-Fisher Scientific) was loaded into exosomes (0.5% loading w/w) by incubation for 10 min in the dark. Curcumin–exosomes were then isolated by ultracentrifugation at 120,000×*g* for 1 h at 15 °C in an SW32 Ti rotor (Optima TL Ultracentrifuge, Beckman Coulter) and resuspended in MilliQ water. Exosomes were also loaded with nucleic acids (random siRNA, DNA-CH or miRNA145) by electroporation as previously described [42]. Briefly, exosome pellet was resuspended in PBS 0.1X and gently mixed with the appropriated µl of siRNA, DNA-CH or miRNA (same loading than for EMNs) in a final volume of 400 μl into 0.4 cm electroporation cuvettes. Exosomes were then electroporated using a Gene Pulser II Electroporator (Bio-Rad), at 300 V and 25 μF of capacitance. Lastly, exosomes were incubated in ice for 30 min to allow the exosome membrane to be fully restored. To get rid of free nucleic acids, exosomes were diluted with cold PBS and isolated again by ultracentrifugation at 120,000×*g* for 90 min at 15 °C in 70.1 Ti rotor (Optima TL Ultracentrifuge).

#### EMNs

Loading with curcumin was accomplished by adding the drug (0.5% loading w/w) to the ethanolic lipidic phase prior injection into the aqueous phase. The suspension was then kept under stirring for 10 min in the dark at RT and ultracentrifuged at 35,000 rpm for 1 h at 15 °C. To associate siRNA and dsDNA to EMNs, the required amount of each nucleic acid was dried up (to eliminate the water content) using miVac DUP with Quattro pump (Genevac) and the organic phase was added, vortexed at 12 rpm for 1 min and injected in the aqueous phase together with the lipids. miR145 and miR-scramble (8 µg) was associated with EMNs by adding directly 8 µg of miRNA in the organic phase and injecting it in the aqueous phase. The formulation was kept 10 min under magnetic stirring.

*Encapsulation efficiencies (%EE)* of the drug and nucleic acids in the nanovesicles were determined indirectly by the difference between the total amount of the theoretical amount added in the sample and the free amount found in the supernatant after isolation by ultracentrifugation. The free drug was detected by measuring the fluorescence curcumin emits (λEx = 420 nm, λEm = 535 nm). Free nucleic acids were detected by using the SYBER Gold solution (λEx = 500 nm, λEm = 550 nm) and the  %EE was calculated following the next equation:

$$\% {\text{EE}} = \left( {{\text{W theoretic}}{-}{\text{W free}}} \right)/{\text{W theoretic}} \times 100.$$

Semi-quantification of miR145 encapsulated in EMNs was performed by agarose gel retention assay. Briefly, EMNs + miR145 were concentrated 10 times by a rotavapor and loaded onto a 2% agarose gel in TAE 1X buffer. Electrophoresis was performed at 100 V for 40 min. MiR145 was visualized by SYBR^®^Gold Nucleic Acid Gel Stain (Invitrogen) by UV transillumination and gel photography. Free miR145 was used as a control.

### EMNs functionalization with proteins

EMNs were functionalized with different proteins, recombinant human integrin α6β4 protein (Vitro), Bovine Serum Albumin (BSA, VWR) and Lysozyme (Sigma-Aldrich) by incubation. Briefly, EMNs were prepared as described above, and proteins were added to the formulation at a ratio 1:100 (protein:lipids) on an orbital shaker for 20 min at RT. To assess the protein association of ITGα6β4, F-EMNs were ultracentrifuged at 60,000 rpm 1 h 15 °C in a Beckman 70.1 Ti rotor (Optima TL Ultracentrifuge) to isolate functionalized EMNs from free protein, and both fractions were loaded on 10% acrylamide gel and detected by fluorescent western blotting using the primary monoclonal antibody ITGβ4 (G-7, Santa Cruz Biotechnology). Physicochemical properties of functionalized EMNs were also measured by DLS and LDA. EMNs + miRNA145 were functionalized with ITGβ4 following the same procedure.

### Specific interaction integrin-laminin

Coverslips were coated with 1 ml (10 µg/ml) of Laminin-V (Cultrex^®^, Vitro) and incubated at 37 °C o/n. Coverslips were then washed twice with PBS 1X and 100 µl of EMNs with/without integrin functionalization (EMNs and F-EMNs) were added and incubated for 24 h at 37 °C. Then, the coverslips were washed again 3 × 5 min with PBS 1X and mounted on slides with 8 μl of Mowiol 4–88 Reagent (Merck, Spain), dried at RT and conserved in the dark at − 20 °C to be analyzed later on by the confocal microscopy.

### miR145 transfection

#### Confocal microscopy analysis

A549 cells were seeded on coverslips in 24-well plate at a density of 8 × 10^4^ cells/well in complete medium. The following day, the same quantity of EMNs or F-EMNs labeled with NBD-CH and loaded with miR145-Cy5 were incubated with the cells in medium without supplement for 4 h in the dark. After incubation, medium was removed, cells were washed with PBS three times and then, fixed with paraformaldehyde (PFA; 4% v/v in PBS) in the dark at RT for 15 min prior to counterstain the nuclei with DAPI for 10 min. Lastly, coverslips were mounted on clean slides with 8 μl of Mowiol, dried at RT, and conserved in the dark at − 20 °C to be analyzed later on by Confocal Laser-Scanning Microscope (CSLM, Leica TCS SP5). Cells were also transfected with miR145-Cy5-loaded exosomes and F-EMNs, adding the same amount of miR145-Cy5 (8 µg) to the cells and following the just mentioned protocol.

#### FACS analysis

miR145-Cy5-loaded EMNs, F-EMNs and exosomes were incubated with the cells as described above. After incubation, the medium was removed, cells were washed with PBS three times and trypsinized. A549 cells incubated with PBS instead of nanovesicles were also included as a control. Collected cells were fixed in 0.4% paraformaldehyde and kept at 4 °C until FACS analysis. The percent of Cy5 positive cells was determined by a FACScan flow cytometer (BD Biosciences). A minimum of 10,000 events per condition was measured. The analysis of the results was performed using FlowJo Software (TreeStar Inc., Ashland, USA).

#### Transfection

24 h before transfection, A549 cells were seeded on 6-well plate at a low density of 25 × 10^4^ cells/well in complete DMEM. The next day, complete medium was removed, cells were washed with PBS 1X and new medium without supplement was added. 8 µg of miR145 associated with EMNs and F-EMNs, as well as miR145 free in solution (as a control) were added to each well and incubated at 37 °C and 5% CO_2_ atmosphere for 4 h. After incubation, the culture medium was removed, cells were washed with PBS and fresh complete medium was added and further incubated for 96 h prior to being collected for analyses by RT-PCR and functional assays (colony forming assay and Western blot).

#### RT-PCR analysis

Total microRNA was extracted from transfected A549 cells using the microRNA Purification Kit (Norgen Biotek Corp.) following the manufacturer’s protocol. After nanodrop RNA quantification, the RNA was retrotranscribed into cDNA using the qScript™ microRNA cDNA Synthesis Kit (Quanta Bioscience™) according to manufacturer’s instructions. Quantitative Real Time-PCR was performed using PerfeCta SYBR Green SuperMix (Quanta Bioscience™) in an AriaMx Real-time PCR System (Agilent Genomics). The relative quantities of miR145 were normalized using the housekeeping RNU-6 and using the comparative CT method. For miRNA quantitation, specific forward, reverse and universal primers were acquired from Eurofins (Fisher Scientific): hsa-miR145-5p (5′-CGCGCGTTCCAGTTTTCCCAGG-3′) and universal reverse PCR primer (5′-GTGCAGGGTCCGAGGT-3′), and the housekeeping small RNA control primer RNU6 (5′-CTCGCTTCGGCAGCACA-3′, 5′-AACGCTTCACGAATTTGCGT-3′). The PCR conditions consisted of 2 min of initial denaturation at 95 °C, 40 cycles of denaturation at 95 °C for 5 s and annealing at 60 °C for 30 s, and lastly, 1 min of activation at 95 °C, annealing at 55 °C for 15 s and elongation at 95 °C for 30 s. Each experiment was performed in triplicate.

### In vivo assays

#### Biodistribution

1 × 10^6^ A549 lung cancer Luc cells, kindly provided by Dr. Anxo Vidal (Santiago de Compostela, Spain), were injected in 0.1 ml PBS into the tail vein of 7-week-old female nu/nu mice (Charles River). The tumor growth was follow-up by in vivo bioluminescence imaging using the Xenogen IVIS (IVISR Lumina II). Mice were anesthetized and injected retro-orbitally with 1.5 mg of d-luciferin (15 mg/ml in PBS), images were taken during 5 min with a Xenogen IVIS (IVISR Lumina II) system coupled to Living Image acquisition and analysis software (Xenogen Corporation). For bioluminescence intensity (BLI) plots, photon flux was calculated as previously described [43]. Measurements were performed once a week starting 1 week after tail vein injection and up to 15 weeks. Mice were randomly divided into two groups and treated with miR145-Cy5-loaded F-EMNs (n = 5) or miR145-Cy5-loaded exosomes (n = 5). Each mouse was injected with a dose of 2.5 µg of miR145-Cy5. After 8 h, mice were sacrificed, and the biodistribution of miR145-Cy5 was quantified by ex vivo fluorescence of different organs, lung + tumor, heart, spleen, kidney, and liver, using the using Xenogen IVIS. In each experiment, mice treated with F-EMNs without miR145-Cy5 were used as a control in order to reduce the background tissue.

#### Confocal analysis in tissue samples

At the end of the experiment, once the mice were sacrificed and the fluorescence quantified, lung tumors were extracted and frozen in OCT (Tissue-Tek, Sakura). The xenografted tumors were stained with DAPI and Ki67 (Rabbit Anti-Human Ki-67 Monoclonal Antibody (Clone SP6), #MAD-020310Q, Master Diagnostica) for the identification of metastatic cells. Images were captured in Cy5 emission on an LSM710 Confocal microscope (Zeiss), analyzed and quantified using Fiji software.

#### Mice handling

The animal handling and the experimental procedures were approved by the internal ethical research and animal welfare committee (IIB, UAM), and by the Local Authorities (Comunidad de Madrid, PROEX424/15) which complied with the European Union (Directive 2010/63/UE) and Spanish Government guidelines (Real Decreto 53/20133).

### Statistics

Statistical analyses were performed with a GraphPad Prism^®^ software (version 6.0c). All data are expressed as mean ± standard deviation (SD). Significant differences between two groups were determined by a Student’s t-test and multiple comparisons among conditions were done using one-way analysis of variance (ANOVA) followed by Bonferroni post hoc test. * (p < 0.05), ** (0.05 > p < 0.001), *** (p < 0.0001) was considered statistically significant. All experiments were performed at least in triplicate.

## Additional file

**Additional file 1.** Supplementary data.

## Data Availability

The datasets used and/or analysed during the current study are available from the corresponding author on reasonable request.

## Abbreviations

EMNs: exosome-mimetic nanosystems
F-EMNs: functionalized exosome-mimetic nanosystem
miR145: microRNA-145
miRscr: microRNA-scramble
integrin α6β4: integrin alpha-6 beta-4
CLSM: Confocal Laser Scanning Microscopy
FACS: fluorescent activated cell sorting
TEM: transmission electron microscopy
NMR: nuclear magnetic resonance
IP: intraperitoneal
RO: retro-orbital
