# Supplementary material for: Exosome-mimetic nanoplatforms for targeted cancer drug delivery
Source: J Nanobiotechnology. 2019 Jul 18;17:85. doi: 10.1186/s12951-019-0517-8 (PMC6637649; doi:10.1186/s12951-019-0517-8)
Supplement: Supplementary file 1 — Additional file 1. Supplementary data. [file 12951_2019_517_MOESM1_ESM.docx]

**ADDITIONAL MATERIAL**

# Additional methods

*Materials*

Phosphatidylcholine (Lipoid E PC) and sphingomyelin (Lipoid E SM) were obtained from Lipoid GmbH (Ludwigshafen, Germany). Cholesterol was purchased from Sigma-Aldrich (Madrid, Spain). C16 Ceramide, NDB-6 Cholesterol, and C11 TopFluor Sphingomyelin, were all purchased in Avanti Polar Lipids (Alabaster, AL, USA). MilliQ^®^ water (Simplicity 185, Millipore, Bedford, USA) was used throughout the study. Ethanol of analytical grade was purchased from VWR (Barcelona, Spain). DiR (DiIC18(7) (1,1′-Dioctadecyl-3,3,3′,3′-Tetramethylindotricarbocyanine Iodide)) and DiD (1,1'-dioctadecyl-3,3,3',3'-tetramethylindotricarbocyanine perchlorate) were acquired from Thermo Fisher Scientific (USA). miRNA-145 (miR145; sense strand 5’-GUCCAGUUUUCCCAGGAAUCCCU-3’, antisense strand 5'-GGAUUCCUGGAAAUACUGUUCU-3'), miRNA-145-Cy5 (miR145-Cy5,), miRNA-scramble (miRscr) and a model siRNA were synthesized by Eurofins Genomics (Ebersberg, Germany). DNA-CH was kindly provided from Ramon Eritja (Nucleic Acids Chemistry Group, Institute for Advanced Chemistry of Catalonia, Barcelona, Spain).

# *Cell culture*

SW480 (ATCC^®^ CCL-228), PC-3 (ATCC^®^ CRL-1435) and A549 (ATCC^®^ CCL-185) cells were grown in Dulbecco’s modified Eagle’s medium (DMEM) high glucose (Gibco, Thermo Fisher Scientific), supplemented with 10% fetal bovine serum (FBS) (Thermo Scientific, Spain), and 1% penicillin/streptomycin (Thermo Scientific, Spain). Cells were maintained at 37 ºC in a 5% CO_2_ humidified atmosphere. Trypsin and Phosphate Buffered Saline (PBS) were purchased from Sigma-Aldrich (St. Louis, USA). All cell lines were tested routinely and confirmed to be mycoplasma-free. The A549 cells were authenticated by STR-profiling according to ATCC guidelines.

*Dynamic Light Scattering (DLS) and Laser Doppler Anemometry (LDA)*

The hydrodynamic diameter, polydispersity index and superficial charge of the exosomes and EMNs were measured using a Zetasizer Nano ZS (Malvern Instruments, UK). Measurements were performed in PBS 1X (exosomes) and MilliQ water (EMNs) at room temperature (RT). For the zeta potential measurements, samples were diluted in 1 mM potassium chloride (KCl).

*Nanoparticle Tracking Analysis (NTA)*

Particle size and concentration distribution of the EMNs and exosomes were also measured using NTA (v2.3; Malvern Instruments, Malvern, UK) according to manufacturer’s instructions. Briefly, EMNs samples were vortexed and diluted to a final dilution of 1:1000 in milliQ H2O and exosomes 1:100. Blank-filtered H_2_O was run as a negative control. Each sample analysis was conducted for 60 s and measured five times using Nanosight automatic analysis settings. The detection threshold was set to level 11 and camera level to 15.

*Stability*

A stability study was performed in human plasma and cell culture media (DMEM supplemented with 1% FBS) incubated at 37 ºC. The colloidal properties of EMNs were determined using the Zetasizer Nano ZS each hour up to 5h for human plasma and 20h for DMEM. Stability of the formulation under storage conditions was also tested in PBS 1X at 4ºC up to 3 months. All measurements were performed in sextuples.

*Nuclear magnetic resonance spectroscopy (NMR)*

NMR experiments relying on ^1^H and ^31^P detection were measured on a Varian Inova 17.6 T spectrometer (750 MHz proton resonance) equipped with a triple resonance HCP probe and z- gradient. The ^13^C NMR spectra were measured on a Varian Mercury 7.04 T (75.4 MHz, ^13^C resonance) equipped with a double resonance ATB probe with z-gradient. The spectra were processed and analyzed with MestreNova software v11.0 (*Mestrelab. inc.*).

*Cryogenic transmission electron microscopy (cryo-TEM)*

Samples were initially vitrified according to Dubochet protocol [43]. Briefly, an aliquot of 3,5 μL of each sample was applied to glow-discharged holey grids for 1 min, blotted, and rapidly plunged into liquid ethane at −180 °C and kept at this temperature until visualization. Images were obtained at 0°-tilt under minimum dose conditions using a field emission gun Tecnai 20 G2 Microscope (FEI, Eindhoven, The Netherlands) equipped with a Gatan cold stage operated at 200 keV. Low-dose images were collected at a nominal magnification of ∼50,000× by using an FEI Eagle CCD camera with a step size of 15μm. The original pixel size of the acquired images was 2.74Å.

*Exosome isolation.*

SW480 (ATCC^®^ CCL-228), PC-3 (ATCC^®^ CRL-1435) and A549 (ATCC^®^ CCL-185) cells were grown in complete DMEM supplemented with 10% FBS and 1% Pen/Strep in a humidified 37ºC incubator with 5% CO_2_. When cells reached a confluency of 80%, cells were washed with phosphate buffer saline (PBS) 1X and cultured in serum-free DMEM (conditioned medium) for 16 hours. Exosomes secreted in the conditioned medium were isolated as previously described with minor modifications [1]. Briefly, the conditioned medium was first centrifuged to remove cells and debris at 300 xg for 10 min and 16,500 xg for 20 min at 4ºC. Then, the supernatant was collected, filtered by a 0,22 μm syringe filter (Pall PharmAssure) and centrifuged at 120,000 xg for 90 min at 4 ºC using a SW 32 Ti Rotor (Optima TL Ultracentrifuge; Beckman Coulter). The exosome pellet was then washed with abundant PBS 1X at 120,000 xg for 70 min at 4ºC. Exosomes from plasma were isolated following the same protocol with a slight modification due to plasma viscosity and protein and lipid abundance compared with the cell supernatant. Briefly, venous blood samples were collected in lavender-top tubes of 10 mL with EDTA (BD VacutainerTM K2E) and were centrifuged at 2,000 xg for 15 min at 4ºC, then, plasma was collected and centrifuged at 500 xg for 30 min at 4ºC to remove cells and at 12,000 xg for 45 min at 4ºC to sediment cell debris and macrovesicles. Next, supernatant was filtered through a 0.22 um syringe filter to remove particles larger than 200 nm and diluted in PBS 1X (1:6). After that, sample was ultracentrifuged at 120,000 xg for 90 min at 4ºC. The exosome pellet was washed in a large volume of PBS 1X (36 mL), ultracentrifuged at 120,000 xg for 70 min at 4ºC. This step was repeated one more time (wash and centrifuge). Exosome pellets from cells and plasma were finally resuspended in 50 μL of the appropriated buffer (PBS 1X for DLS measurements and TEM or in RIPA 1X buffer for protein quantification and western blotting) and immediately used for experiments or stored at -80 ºC for later analysis.

*Western Blot analysis.*

The protein level of the isolated exosomes was determined using the microBCA protein assay kit according to the manufacturer’s instructions (Thermo Scientific, Spain). Western blot analyses were performed using standard techniques. Total exosomal protein (16 μg per lane) was run on 10% SDS-PAGE gel and transferred to polyvinylidene fluoride (PVDF) membranes. After blocking and washing, the membranes were incubated with antibodies against Alix (3A9, NOVUS, 1:200) and CD9 (C-4, Santa Cruz Biotechnology, 1:200) in TTBS-5% milk overnight at 4 ºC. After that, membranes were incubated with the secondary antibody Peroxidase AffiniPure Goat Anti-Mouse IgG (H+L) (114094, Jackson ImmunoResearch^®^) and proteins were detected using the enhanced chemioluminescence ECL kit according to the manufacturer’s protocol (Thermo Scientific, Spain).

*Transmission Electron Microscopy (TEM).*

Morphology and structure of the nanovesicles were observed by TEM (JEOL JEM-2010). 10 μL of the sample was loaded on the grid, incubated for 3 min at room temperature (RT) and stained in 10 μL of 2% phosphotungstic acid solution for 1 min. The excess of phosphotungstic solution was removed with filter paper and the grid was washed seven times in a water droplet for 2-3 sec and then air-dried overnight.

*Protein identification of exosomes by LC-MS/MS.*

Exosomes from A549 cells were loaded on a 10% SDS-PAGE gel. The run was stopped as soon as the front had penetrated 3 mm into the resolving gel [2,3]. The protein band was detected by Sypro-Ruby fluorescent staining (Lonza, Switzerland), excised, and processed for in-gel, manual tryptic digestion as described elsewhere [4]. Peptides were extracted by carrying out three 20-min incubations in 40 μL of 60% acetonitrile dissolved in 0.5% HCOOH. The resulting peptide extracts were pooled, concentrated in a SpeedVac, and stored at −20 °C.

*Mass spectrometric analysis.*

Digested peptides were separated using Reverse Phase Chromatography. Gradient was created using a micro liquid chromatography system (Eksigent Technologies nanoLC 400, SCIEX) coupled to high speed Triple TOF 6600 mass spectrometer (SCIEX) with a micro flow source. The chosen analytical column was a silica-based reversed phase column YMC-TRIART C18 150 × 0.30 mm, 3 mm particle size and 120 Å pore size (YMC Technologies, Teknokroma). The trap column was a YMC-TRIART C18 (YMC Technologies, Teknokroma with a 3 mm particle size and 120 Å pore size, switched on-line with the analytical column. The loading pump delivered a solution of 0.1% formic acid in water at 10 µl/min. The micro-pump generated a flow-rate of 5 µl/min and was operated under gradient elution conditions, using 0.1% formic acid in water as mobile phase A, and 0.1% formic acid in acetonitrile as mobile phase B. Peptides were separated using a 90 minutes gradient ranging from 2% to 90% mobile phase B (mobile phase A: 2% acetonitrile, 0.1% formic acid; mobile phase B: 100% acetonitrile, 0.1% formic acid). Injection volume was 4 µl.

Data acquisition was performed in a TripleTOF 6600 System (SCIEX, Foster City, CA) using a Data dependent workflow. Source and interface conditions were the following: ionspray voltage floating (ISVF) 5500 V, curtain gas (CUR) 25, collision energy (CE) 10 and ion source gas 1 (GS1) 25. Instrument was operated with Analyst TF 1.7.1 software (SCIEX, USA). Switching criteria was set to ions greater than mass to charge ratio (m/z) 350 and smaller than m/z 1400 with charge state of 2–5, mass tolerance 250ppm and an abundance threshold of more than 200 counts (cps). Former target ions were excluded for 15 s. The instrument was automatically calibrated every 4 hours using as external calibrant tryptic peptides from PepCalMix.

*Data Analysis.*

After MS/MS analysis, data files were processed using ProteinPilotTM 5.0.1 software from Sciex which uses the algorithm ParagonTM for database search and ProgroupTM for data grouping. Data were searched using a Human specific Uniprot database. False discovery rate was performed using a non-lineal fitting method displaying only those results that reported a1% Global false discovery rate or better [5]. Functional analysis was performed by FunRich open access software (Functional Enrichment analysis tool) for functional enrichment and interaction network analysis (http://funrich.org/index.html). For statistics, FunRich use hypergeometric test, BH and Bonferroni [6,7].

*Cellular uptake of EMNs*

Uptake was investigated by using fluorescent-labeled EMNs (TopFluor-SM) by means of a Confocal Laser-Scanning Microscope (CSLM; Leica TCS SP5). Cancer cell lines (SW480, A549 and PC3) were grown on coverslips in a P24 plate at a density of 10^5^ cells/well for 24 hours at 37ºC. Fluorescent–labeled EMNs were added in fresh medium without supplements to cultured cells and incubated for 4 hours in the dark. After incubation, cells were rinsed with PBS 1X (pH 7.4) three times and then were treated with paraformaldehyde (PFA; 4% v/v in PBS) in the dark at RT for 15 minutes. Cells were washed again with PBS at least three times, and then the nuclei were counterstained with Di Aminido Phenyl Indol (DAPI; Cell Search, Sigma-Aldrich) or Hoechst (Thermo Scientific) for 3 minutes in the dark at RT. Finally, cells were washed again, and coverslips were mounted on clean slides with 8 μL of Mowiol (Calbiochem^®^), dried at RT, and conserved in the dark at -20ºC to be analyzed later on by the confocal microscopy. The three-dimensional (3D) structure of the cells treated with EMNs-NBD+miR145-Cy5 was reconstructed from corresponding confocal images using Leica Application Suite X (LAS X) software.

# *Cytotoxicity assay*

The cytotoxicity of exosomes and EMNs were assessed by the 3-(4,5-dimethylthiazol-2-yl)-2,5-diphenyltetrazolium bromide (MTT, Thermo Fisher Scientific) assay. Briefly, cells were seeded at a density of 10^4^ cells/well in a 96-well plate containing 100 μL of fresh culture medium and incubated overnight to allow cell attachment for subsequent study. Then, cells were cultured in the presence of different concentrations of exosomes/EMNs and for 48h at 37ºC. After the incubation, MTT (5 mg/mL) was added to medium and further incubated for 4 h, then 100 μL DMSO was added to dissolve the formazan crystals formed in the live cells for 10 min at 37ºC. The absorbance at 570 nm was recorded using a spectrometer.

# *Immunofluorescence protocol*

A549 cells were seeded onto coverslips in 24-well plate and incubated overnight at 37 ºC. The following day, cells were washed with PBS twice and fixed with PFA 4% for 15 min at RT. After two more washing steps, cells were permeabilized with 0.2% triton x-100 for 10 min at RT, washed again, and subsequently incubated with PBS + 3% BSA for 1h at RT. Next, cells were incubated with the primary monoclonal antibody Laminin-5 (1:50, Dako) in BSA 2% for 1h at RT, washed with PBS 1X 3x5 min and for visualization, DyLight^TM^ 680 conjugated secondary antibody (35518, Thermo Fisher Scientific) was used. Coverslips were washed again with PBS 1X and treated with DAPI for cell nuclei staining. Lastly, coverslips were mounted on slides with 8 μL of Mowiol, dried at RT and conserved in the dark at -20ºC to be analyzed later on by the confocal microscopy.

# *Perfusion of EMNs in a 3D model of tumor cells and immunofluorescence.*

The 3D model was performed as previously reported [8]. Briefly, 1 x 10^6^ cells were added in a 50 µL drop on the surface of the 3D polystyrene scaffolds (Alvetex^®^, Reprocell Europe) and incubated for 5h at 37ºC. After that, 10 mL of fresh culture medium was added to the P6 well plate. Then, the scaffold was placed inside a bioreactor and coupled to an automatic syringe pump (New Era Pump Systems, Inc.). EMNs were diluted in fresh culture medium and perfused under dynamic conditions (250 µL/h for 2h) and then, the Scaffold was washed with cold PBS (500 μl/h for 1 h). Finally, the scaffold was removed from the bioreactor and fixed with PFA 4% for 15 min and washed 3x5 min with PBS 1X prior the immunofluorescence protocol. Scaffolds were then incubated with the primary antibody against Laminin-5 (M7262, Dako) for 1h at RT, washed with PBS 1X 3x5 min and for visualization, DyLight^TM^ 680 conjugated secondary antibody (35518, Thermo Fisher Scientific) was used. Scaffolds were washed again with PBS 1X and then treated with DAPI for cell nuclei staining. Scaffolds were analyzed by CLSM at 630x magnification.

# *Labelling of exosomes and EMNs*

Exosomes were labeled with the lipophilic fluorescent DiD, according to Tian et al. protocol [9], with some modifications. Briefly, exosome pellet was resuspended in PBS 1X mixed with DiD (0,5% loading w/w) and incubated for 10 min in the dark. Labeled exosomes were then ultracentrifuged at 200,000 xg for 1 hour at 15ºC in a SW32 Ti rotor (Optima TL Ultracentrifuge, Beckman Coulter) to remove free DiD and resuspended in PBS 1X. EMNs were labelled with different fluorophores (DiD, NBD, TopFluor, DiR, Nile Red and Cy5) separately or in combination, by adding them to the organic phase together with the lipids and injecting them into the stirred aqueous phase. The suspension was kept under stirring for 10 min in the dark at RT. EMNs were then ultracentrifuged at 30.000 rpm for 1 h at 15ºC in a Beckman 70.1 Ti rotor (Optima TL Ultracentrifuge). Association efficacy (%AE) was determined, in all cases, indirectly by the difference between the total amounts of the theoretical fluorophore added in the sample and the free fluorophore found in the supernatant after isolation. Free fluorophore intensity was detected by EnVision multilabel plate reader (Perkin Elmer, Whaltam, MA, USA), DiD (λEx = 644 nm, λEm = 665 nm), NBD (λEx = 466 nm, λEm = 535 nm), TopFluor (λEx = 495 nm, λEm = 503 nm), DiR (λEx = 750 nm, λEm = 780 nm), Nile Red (λEx = 553 nm, λEm = 610 nm), Cy5 (λEx = 647 nm, λEm = 665nm), and the % of association efficiency (AE) was calculated following the next equation: %AE = (W theoretic – W free) / W theoretic x 100.

*Colony forming assay.*

After 4 hours of transfection with EMNs, transfected A549 cells were seeded at 400, 600 and 800 cells per well in a 12 well plate. Cell colonies were allowed to grow for 6 days in complete DMEM medium. Colonies were then stained with MTT solution (5 mg/ml) for 3 hours, washed with PBS 1X and air dried. The number of colonies per well was determined by imaging and analyzed with Image J software. All experiments were carried out in triplicate.

*Chloroquine treatment.*

After transfection, 600 cells were seeded in a 12-well plate in complete DMEM medium and treated with 40 µM chloroquine (Sigma-Aldrich) overnight. The next day, cells were washed with PBS 1X and fresh culture medium was added.

*N-Cadherin downregulation by WB.*

Protein levels of transfected cells and controls were determined by DC^TM^ protein assay (Bio-Rad Laboratories). Equivalent levels of proteins were separated by SDS-PAGE and transferred to a PVDF membrane and incubated with primary monoclonal antibodies against N-Cadherin (1:500, 3B9, #33-3900, Life Technologies) and ß-actin (1:2000, T6199, Sigma-Aldrich). The ECL method (Thermo Scientific) was used to visualize the expression of proteins.

*Comparative biodistribution of two different administration routes.*

Once the tumors were visible by bioluminescence, mice were treated with EMNs + miR145-Cy5 injected by retro-orbital inoculation (n=3) or by intraperitoneal route (n=3). 8 hours later, mice were sacrificed and biodistribution of miR145-Cy5 quantified by *ex vivo* fluorescence of different organs, lung + tumor, heart, spleen, kidney and liver, using Xenogen IVIS (IVISR Lumina II). In each experiment, mice treated with EMNs without miR145-Cy5 were used as control in order to reduce the background tissue.

*Association efficiency of BSA and lysozyme to EMNs*

BSA and lysozyme (LYS) association to EMNs was measured by using Amicon Ultra – 0,5 mL centrifugal filters (100 K) (Merk Millipore) at 2700 xg for 20 min to separate the free protein from the liposomes. The association efficiency of the proteins to EMNs was calculated by measuring the concentration of free protein using Quick Start Bradford 1X Dye Reagent (BioRad), reading the absorbance at 595 nm in a spectrophotometer (Tecan Infinite M1000), and following the next equation:

%AE = (C_theoretic protein_ – C_free protein_) / C_theoretic protein_ x 100%

where C is concentration in mg/mL.

**Additional references**

[1] C. Théry, S. Amigorena, G. Raposo, A. Clayton, Isolation and characterization of exosomes from cell culture supernatants and biological fluids., Curr. Protoc. Cell Biol. Chapter 3 (2006) Unit 3.22. doi:10.1002/0471143030.cb0322s30.

[2] E. Bonzon-Kulichenko, D. Pérez-Hernández, E. Núñez, P. Martínez-Acedo, P. Navarro, M. Trevisan-Herraz, M. del Carmen Ramos, S. Sierra, S. Martínez-Martínez, M. Ruiz-Meana, E. Miró-Casas, D. García-Dorado, J.M. Redondo, J.S. Burgos, J. Vázquez, A Robust Method for Quantitative High-throughput Analysis of Proteomes by ^18^ O Labeling, Mol. Cell. Proteomics. 10 (2011) M110.003335. doi:10.1074/mcp.M110.003335.

[3] D. Perez-Hernandez, C. Gutiérrez-Vázquez, I. Jorge, S. López-Martín, A. Ursa, F. Sánchez-Madrid, J. Vázquez, M. Yáñez-Mó, The intracellular interactome of tetraspanin-enriched microdomains reveals their function as sorting machineries toward exosomes., J. Biol. Chem. 288 (2013) 11649–61. doi:10.1074/jbc.M112.445304.

[4] A. Shevchenko, M. Wilm, O. Vorm, M. Mann, Mass spectrometric sequencing of proteins silver-stained polyacrylamide gels., Anal. Chem. 68 (1996) 850–8. http://www.ncbi.nlm.nih.gov/pubmed/8779443 (accessed January 9, 2019).

[5] I. V Shilov, S.L. Seymour, A.A. Patel, A. Loboda, W.H. Tang, S.P. Keating, C.L. Hunter, L.M. Nuwaysir, D.A. Schaeffer, The Paragon Algorithm, a next generation search engine that uses sequence temperature values and feature probabilities to identify peptides from tandem mass spectra., Mol. Cell. Proteomics. 6 (2007) 1638–55. doi:10.1074/mcp.T600050-MCP200.

[6] M. Pathan, S. Keerthikumar, C.-S. Ang, L. Gangoda, C.Y.J. Quek, N.A. Williamson, D. Mouradov, O.M. Sieber, R.J. Simpson, A. Salim, A. Bacic, A.F. Hill, D.A. Stroud, M.T. Ryan, J.I. Agbinya, J.M. Mariadason, A.W. Burgess, S. Mathivanan, FunRich: An open access standalone functional enrichment and interaction network analysis tool, Proteomics. 15 (2015) 2597–2601. doi:10.1002/pmic.201400515.

[7] M. Pathan, S. Keerthikumar, D. Chisanga, R. Alessandro, C.-S. Ang, P. Askenase, A.O. Batagov, A. Benito-Martin, G. Camussi, A. Clayton, F. Collino, D. Di Vizio, J.M. Falcon-Perez, P. Fonseca, P. Fonseka, S. Fontana, Y.S. Gho, A. Hendrix, E.N.-’t Hoen, N. Iraci, K. Kastaniegaard, T. Kislinger, J. Kowal, I. V Kurochkin, T. Leonardi, Y. Liang, A. Llorente, T.R. Lunavat, S. Maji, F. Monteleone, A. Øverbye, T. Panaretakis, T. Patel, H. Peinado, S. Pluchino, S. Principe, G. Ronquist, F. Royo, S. Sahoo, C. Spinelli, A. Stensballe, C. Théry, M.J.C. van Herwijnen, M. Wauben, J.L. Welton, K. Zhao, S. Mathivanan, A novel community driven software for functional enrichment analysis of extracellular vesicles data., J. Extracell. Vesicles. 6 (2017) 1321455. doi:10.1080/20013078.2017.1321455.

[8] M. Alonso-Nocelo, R. Abellan-Pose, A. Vidal, M. Abal, N. Csaba, M.J. Alonso, R. Lopez-Lopez, M. de la Fuente, Selective interaction of PEGylated polyglutamic acid nanocapsules with cancer cells in a 3D model of a metastatic lymph node, J. Nanobiotechnology. 14 (2016) 1–9. doi:10.1186/s12951-016-0207-8.

[9] T. Tian, Y. Wang, H. Wang, Z. Zhu, Z. Xiao, Visualizing of the cellular uptake and intracellular trafficking of exosomes by live-cell microscopy, J. Cell. Biochem. 111 (2010) 488–496. doi:10.1002/jcb.22733.

[10] A. Llorente, T. Skotland, T. Sylvänne, D. Kauhanen, T. Róg, A. Orłowski, I. Vattulainen, K. Ekroos, K. Sandvig, Molecular lipidomics of exosomes released by PC-3 prostate cancer cells, Biochim. Biophys. Acta - Mol. Cell Biol. Lipids. 1831 (2013) 1302–1309. doi:10.1016/j.bbalip.2013.04.011.

**Additional tables**

**Table S1. Different possible compositions of EMNs.**

| Composition | Ratio (w/w) | Total mass (mg) | Size (nm) | PdI |
| --- | --- | --- | --- | --- |
| PC:CH | 2:1 | 4.5 | 84 ± 8 | 0.1 |
| PC:CH | 1.5:1 | 2.5 | 89 ± 2 | 0.1 |
| PC:CH:SM | 0.9:1:0.4 | 1 | 87 ± 2 | 0.1 |
| PC:CH:SM | 0.9:1:0.4 | 2 | 134 ± 1 | 0.1 |
| PC:CH:SM:Cer | 0.9:1:0.4:0.03 | 2.02 | 100 ± 8 | 0.2 |
| PC:CH:SM:PE:Cer | 0.4:1:0.4:0.2:0.03 | 0.6 | 98 ± 2 | 0.2 |
| PC:CH:SM:PE:Cer | 0.4:1:0.4:0.2:0.03 | 0.9 | 109 ± 1 | 0.1 |
| PC:CH:SM:PE:Cer | 0.4:1:0.4:0.2:0.03 | 1.6 | 126 ± 3 | 0.1 |
| PC:CH:SM:PE:Cer | 0.4:1:0.4:0.2:0.03 | 2 | 147 ± 2 | 0.1 |
| PC:CH:SM:Cer:DC-CH | 0.4:1:0.4:0.2:0.03:0.04 | 2.07 | 102 ± 3 | 0.2 |
| PC:CH:SM:Cer:DC-CH | 0.4:1:0.4:0.2:0.03:0.07 | 2.09 | 147 ± 5 | 0.2 |
| PC:CH:SM:Cer:ST | 0.4:1:0.4:0.2:0.03:0.002 | 2.03 | 74 ± 1 | 0.3 |
| PC:CH:SM:Cer:CTAB | 0.4:1:0.4:0.2:0.03:0.005 | 2.033 | 91 ± 3 | 0.3 |
| PC:CH:SM:Cer:DOTAP | 0.4:1:0.4:0.2:0.03:0.01 | 2.038 | 67 ± 4 | 0.4 |

**Table S2. Physicochemical properties of EMNs after association of proteins.**

| Protein name | MW (KDa) | pI | Size (nm) | PdI | ZP (mV) |
| --- | --- | --- | --- | --- | --- |
| BSA | 66 | 5.6 | 112 ± 1 | 0.2 | -1 ± 1 |
| Lysozyme | 16.5 | 9.4 | 118 ± 8 | 0.2 | - 4 ± 1 |
| Integrin α6β4 | 188.8 | 5.3 | 110 ± 2 | 0.3 | - 6 ± 1 |

**Abbreviations:** BSA, bovine serum albumin; MW, molecular weight; pI, isoelectric point; PdI, Polydispersity index; ZP, Zeta potential.

**Additionals figures**

# Figure S1. Characterization of isolated isolated exosomes. (a) Western Blot analysis for exosome markers (Alix and CD9) of exosomes from different sources (human plasma and cancer cell lines). (b) Venn diagram illustrating common proteins identified in A549-exosomes (1% FDR) with EV vesiclepedia database and Exocarta database top 100 proteins. (c) The Gene Ontology analysis of the proteins identified by cellular component was performed using FunRich tool. (d) Representative TEM images of exosomes. Scale bars represent 200nm (left) and 50 nm (right) (e) Yield of exosomes from conditioned medium of A549 cell line.


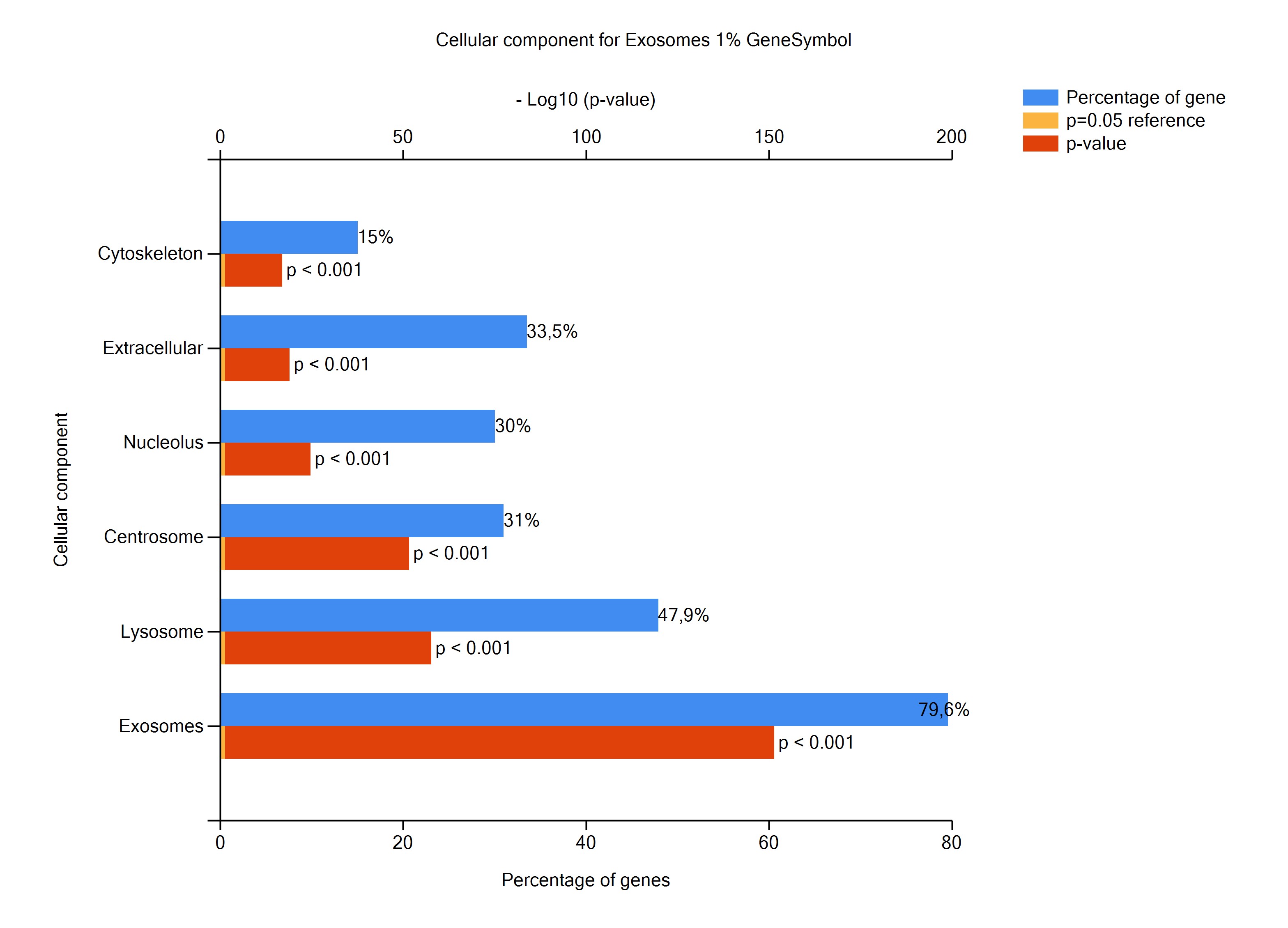

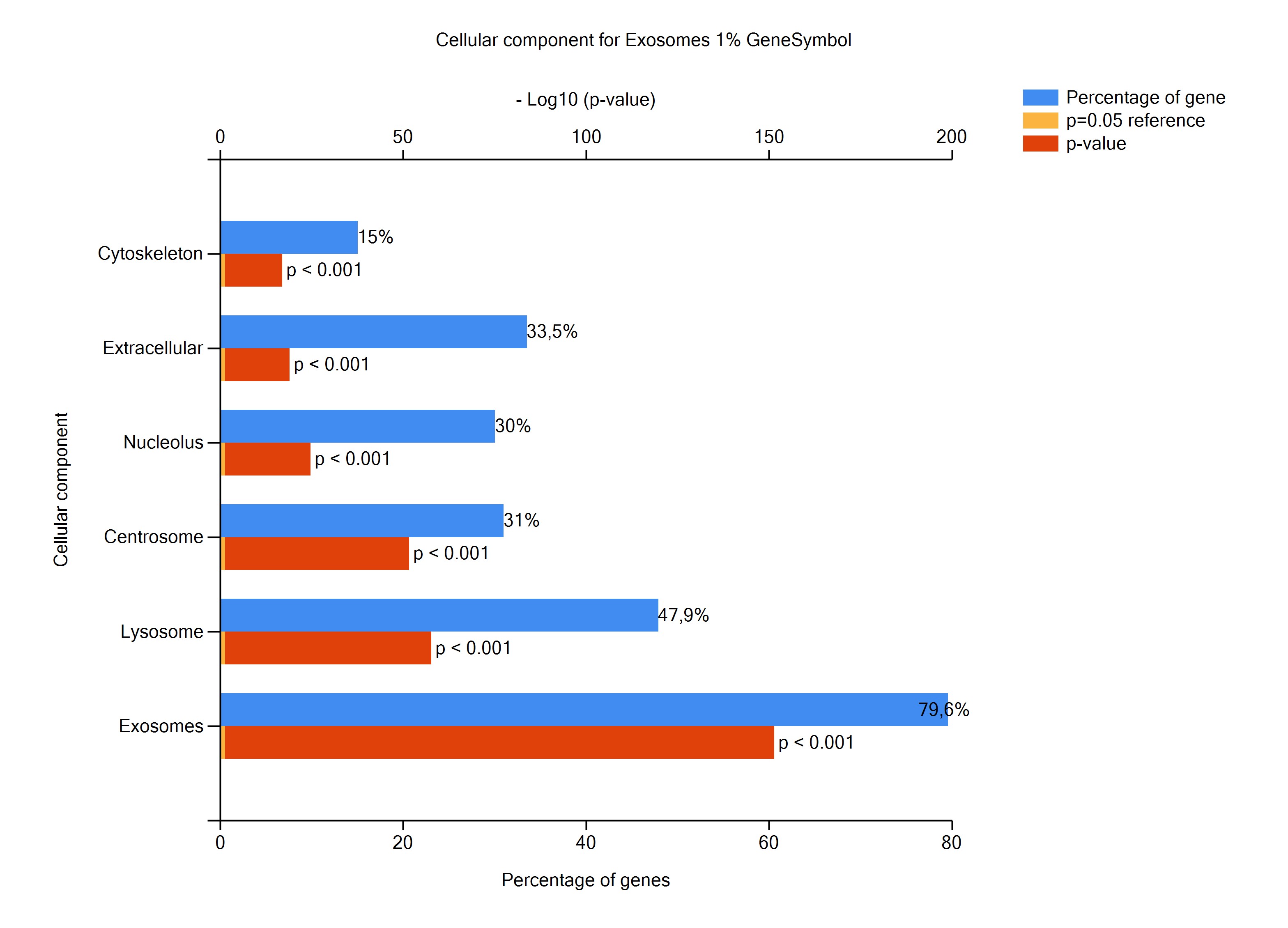

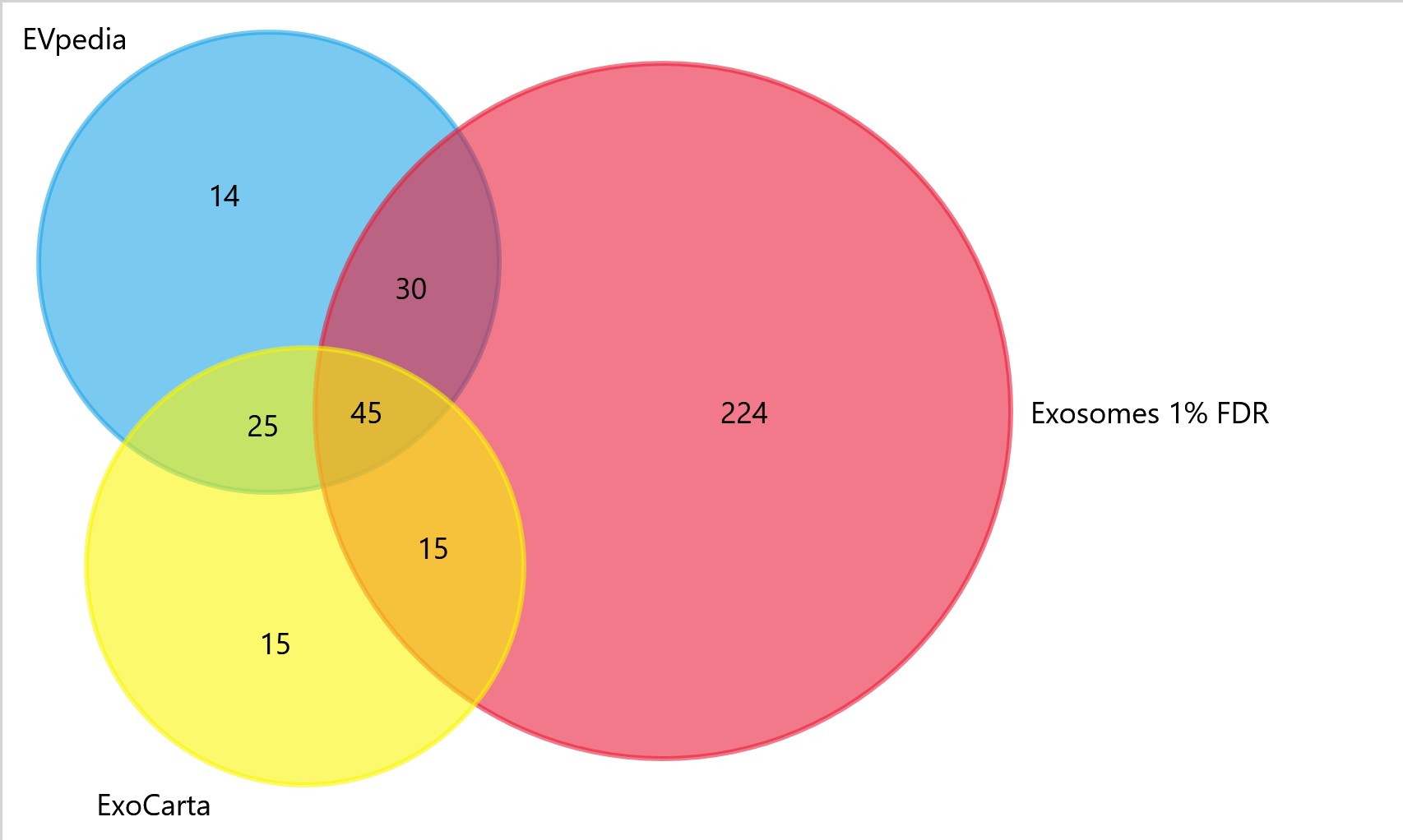


**A549 Exosomes**

TSG101

CD81

CD63

CD9

Alix

…

ExoCarta

Top100

EVpedia

Top100


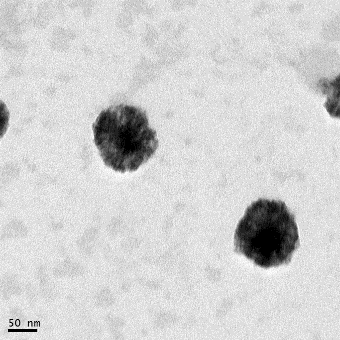

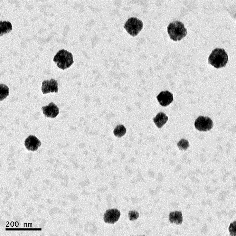

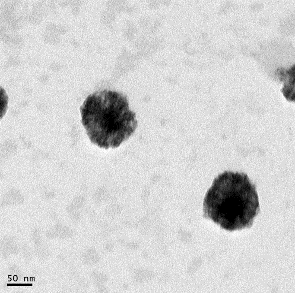


**a**

**b**

**c**

**d**

**e**


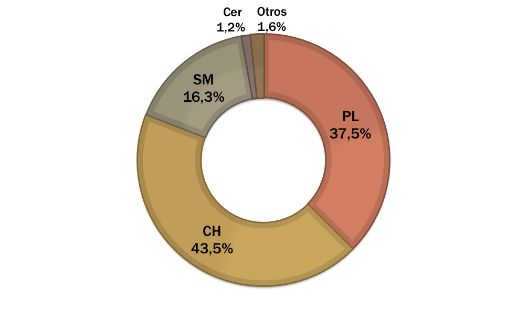


**a**

**b**

**c**

SW480

PC-3

A549

**d**

**e**


**Figure S2. EMNs preparation and *in vitro* characterization. (a)** Lipidomic assay performed by Llorente *et al*. [10] showing a comprehensive and quantitative profiling of lipid species in natural PC-3 exosomes that was followed for the engineering of EMNs. **(b)** Schematic illustration of the ethanol injection method for the EMNs preparation. **(c)** Illustration of exosome-mimetic EMNs and its final lipid composition. **(c)** Cell viability assay measured by MTT of EMNs after 48h incubation in different cancer cell lines (PC-3, prostate cancer; SW480, colon cancer; A549, lung cancer). **(d)** Confocal images showing the effective interaction and internalization of EMNs (green signal) by different cancer cell lines (PC-3, SW480 and A549). Blue channel: nuclei (Hoechst); green channel: EMNs (TopFluor). Scale bars represent 50 µm.

**Figure S3. ^31^P NMR spectra of intact EMNs (upper) and broken EMNs (lower).** In the broken EMNs the NMR integral ratio is close to the theoretical ratio used for the preparation of the sample 0.44:1.

**Figure S4. Protein association efficiency to EMNs.** BSA: Bovine serum albumin, LYS: lysozyme.

Nuclei

**b**

F-EMNs

Laminin-5

Merge


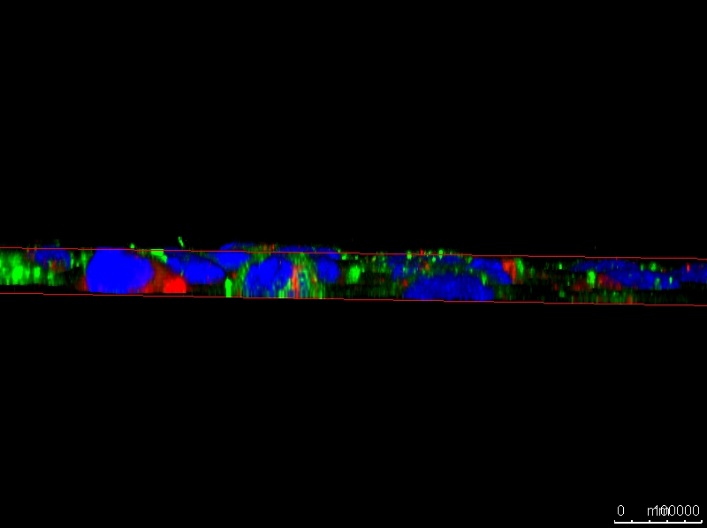

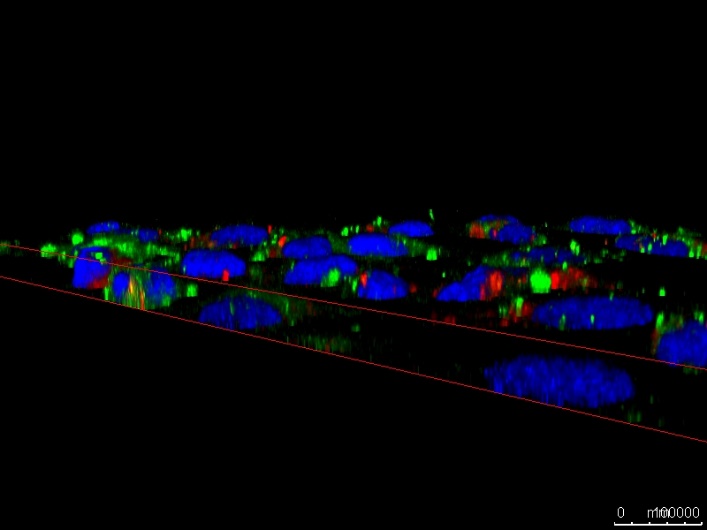


**c**


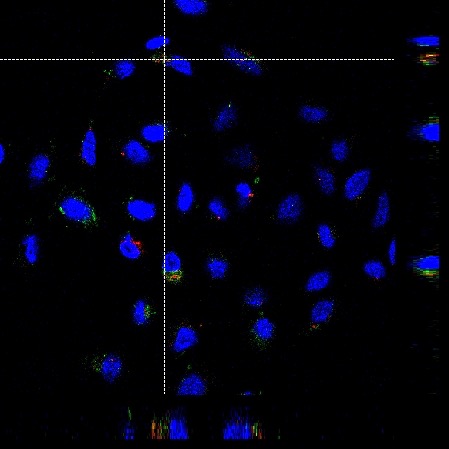

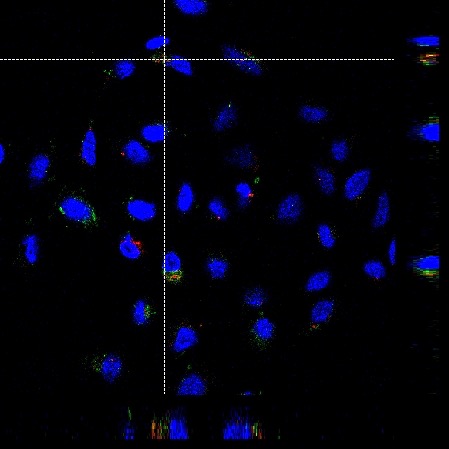

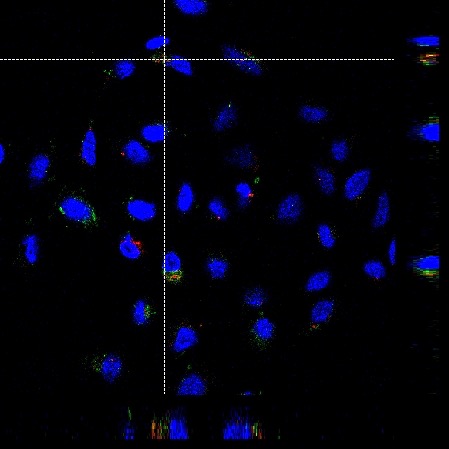


**d**

**a**

Nuclei

Laminin-5

Merge

**Figure S5.** **Confocal microscopy images. (a)** Immunofluorescence staining of laminin-5 (ITGα6β4 receptor) in A549 cells. Blue channel: nuclei (DAPI), red channel: laminin-5 (DyLight 680). Scale bars represent 50 µm. **(b)** 3D culture in A549 lung cell line upon perfusion of F-EMNs, perfusion rate of 250 µl/h x 2h. Blue channel: nuclei (DAPI); green channel: F-EMNs (NBD); red channel: Laminin-5 (DyLight 680). Scale bars represent 75 µm. **(c)** 3D reconstruction of confocal analysis of F-EMNs+miR145 showing the colocalization of F-EMNs (green) with miR145 (red) inside the cells and **(d)** Z-stack projection with orthogonal cross showing red and green spots inside the cells. Blue channel: nuclei (DAPI); green channel: F-EMNs (NBD); red channel: miR145 (Cy5).

Top-Fluor

(495-503 nm)

miRNA - Cy5

(649-666 nm)

DiR

(750-780 nm)


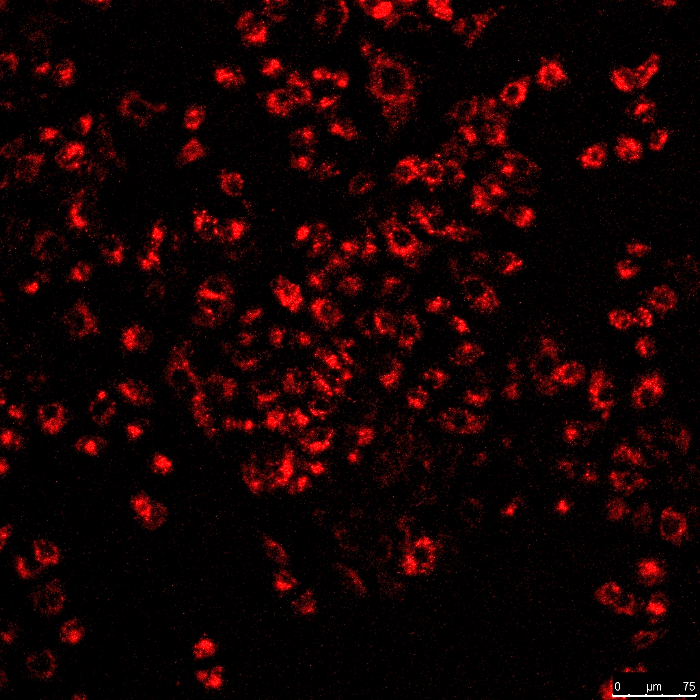

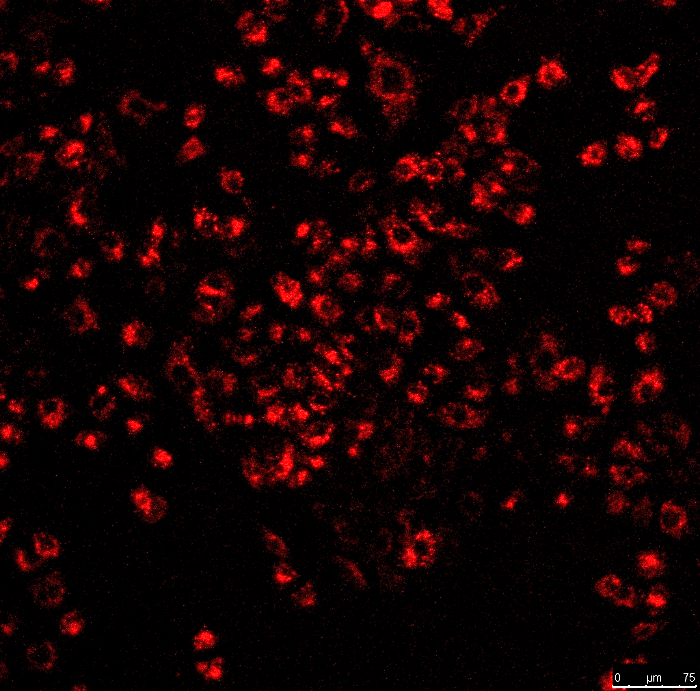

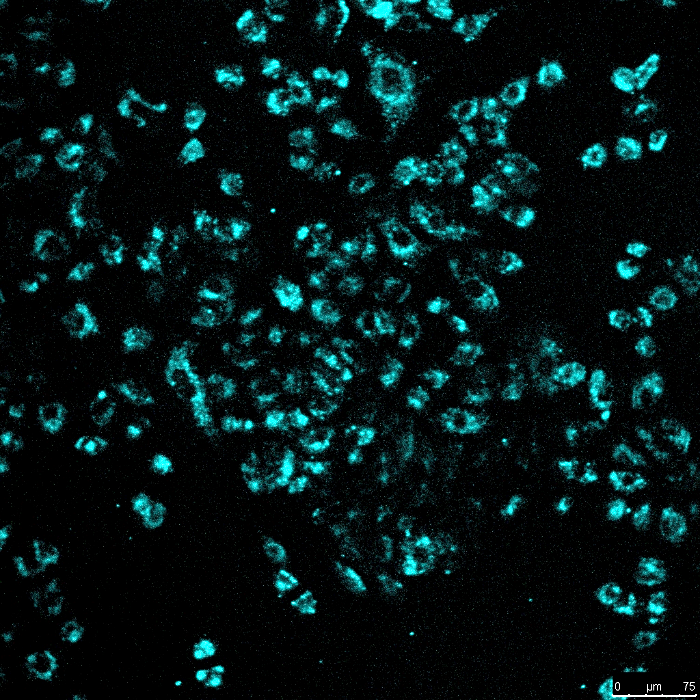

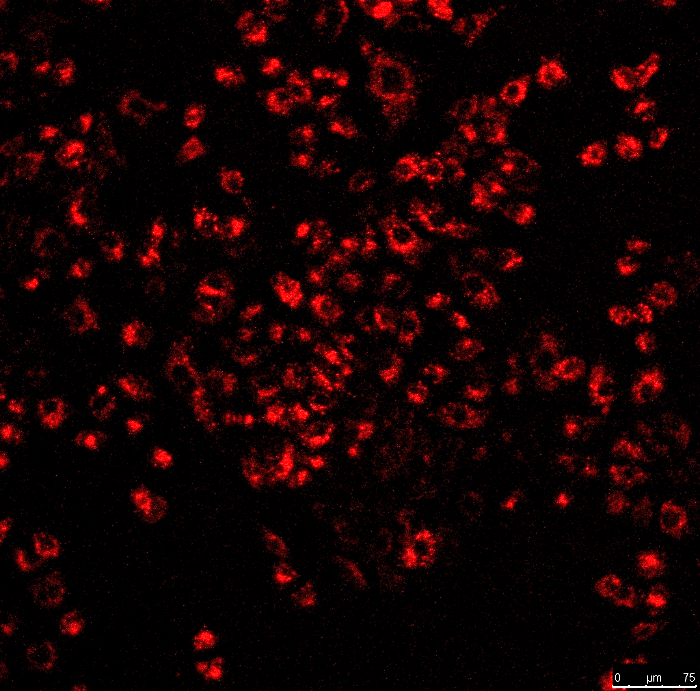

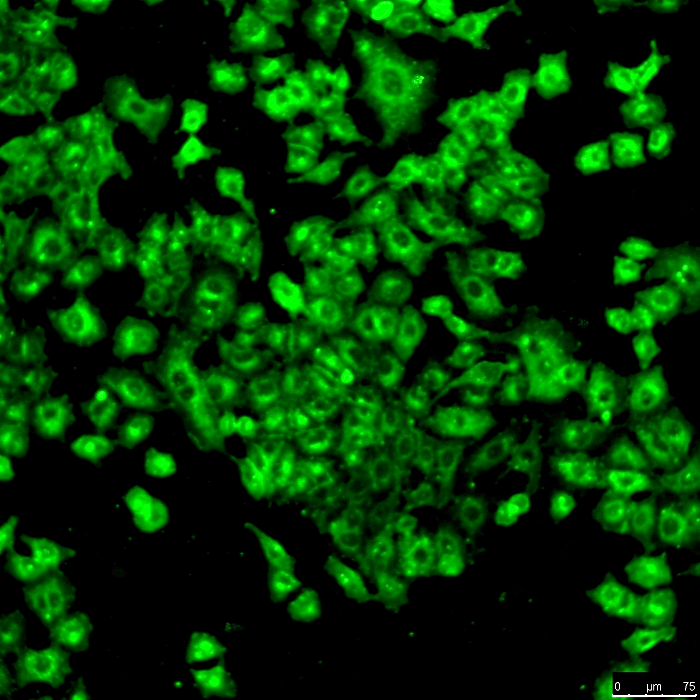

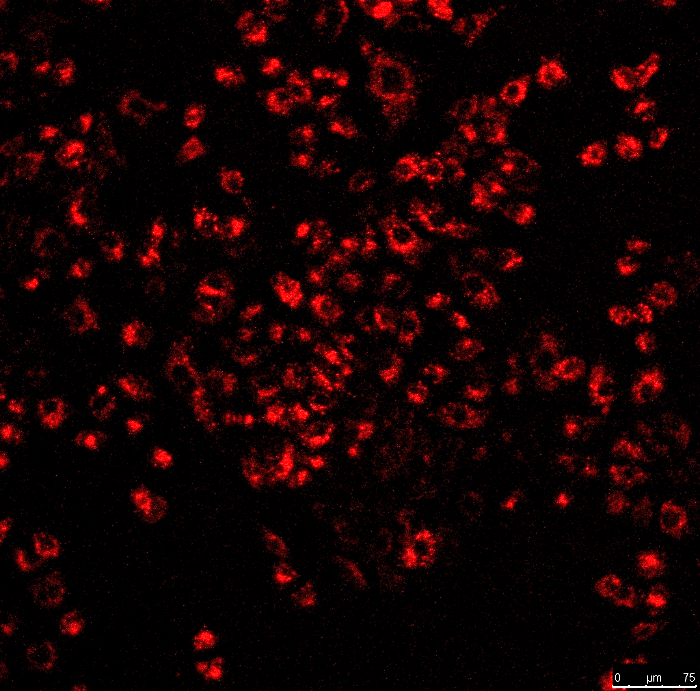


**a**

**b**


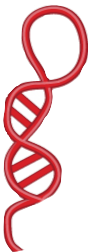


miRNA-145-Cy5

COMPONENTS

FLUOROPHORES

**Figure S6. Triple fluorescent labelling of EMNs.** **(a)** Schematic representation of the triple labelling of EMNs where SM-TopFluor was incorporated into EMNs that encapsulate miR145-Cy5 molecules and EMNs membrane was labelled with the lipophilic dye DiR. **(b)**. Confocal images of triple labelled EMNs internalized by A549 cells. Green channel: EMNs (TopFluor); blue channel: miRNA145 (Cy5); red channel: EMNs (DiR). Scale bars represent 75 µm.

**a**

**b**

**c**


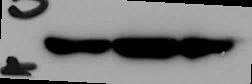


**N – Cadherin**

**β – actin**


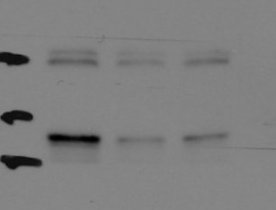


140 KDa **—**

95 KDa **—**

72 KDa **—**

42 KDa **—**

**Control**

**F-EMN+miR145**

**Figure S7.** **Efficient delivery of functionally active miR145 by functionalized EMNs. (a)** Colony forming assay of A549 cells transfected with F-EMNs+miR145 compared to A549 control starting from 400, 600 or 800 initial cells. **(b)** Colony forming assay with and without the lysosomal disruption agent (chloroquine) **(c)** Western blot result showing down regulation of A549 when transfected with EMNs compared to control (untreated cells). (*p value <0,001).


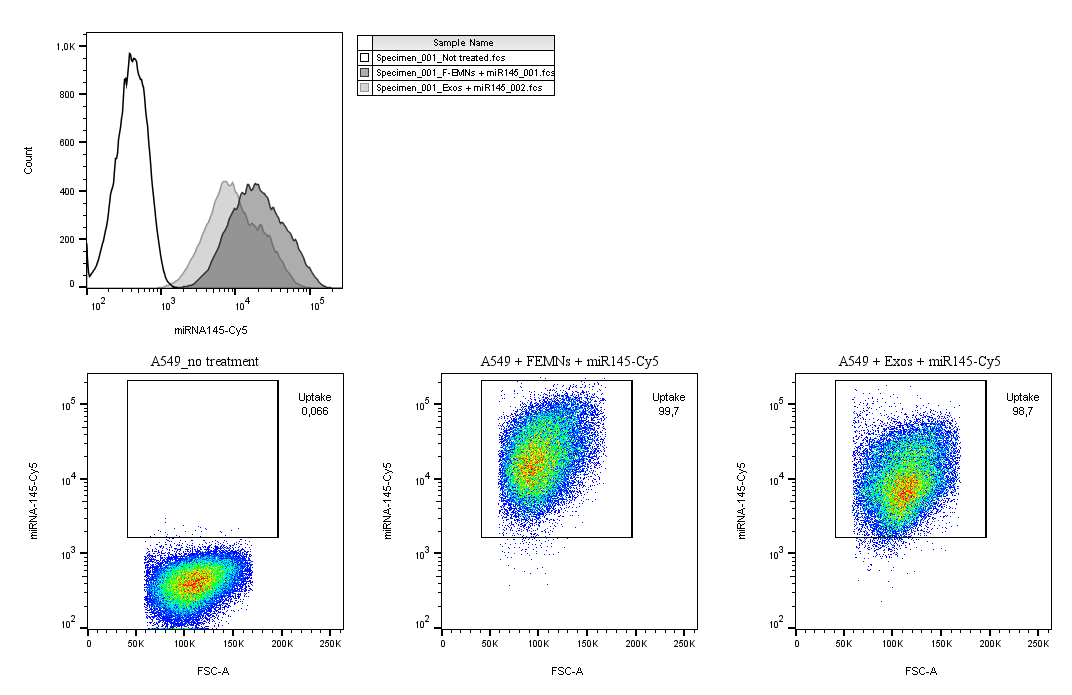


**Figure S8. Cellular uptake of miRNA-145-Cy5 encapsulated F-EMNs and exosomes into A549 cells after 4h incubation.** Comparison of the uptake efficiency of F-EMNs (99,7%) and exosomes (98,7%) by pseudocolor plot.

**
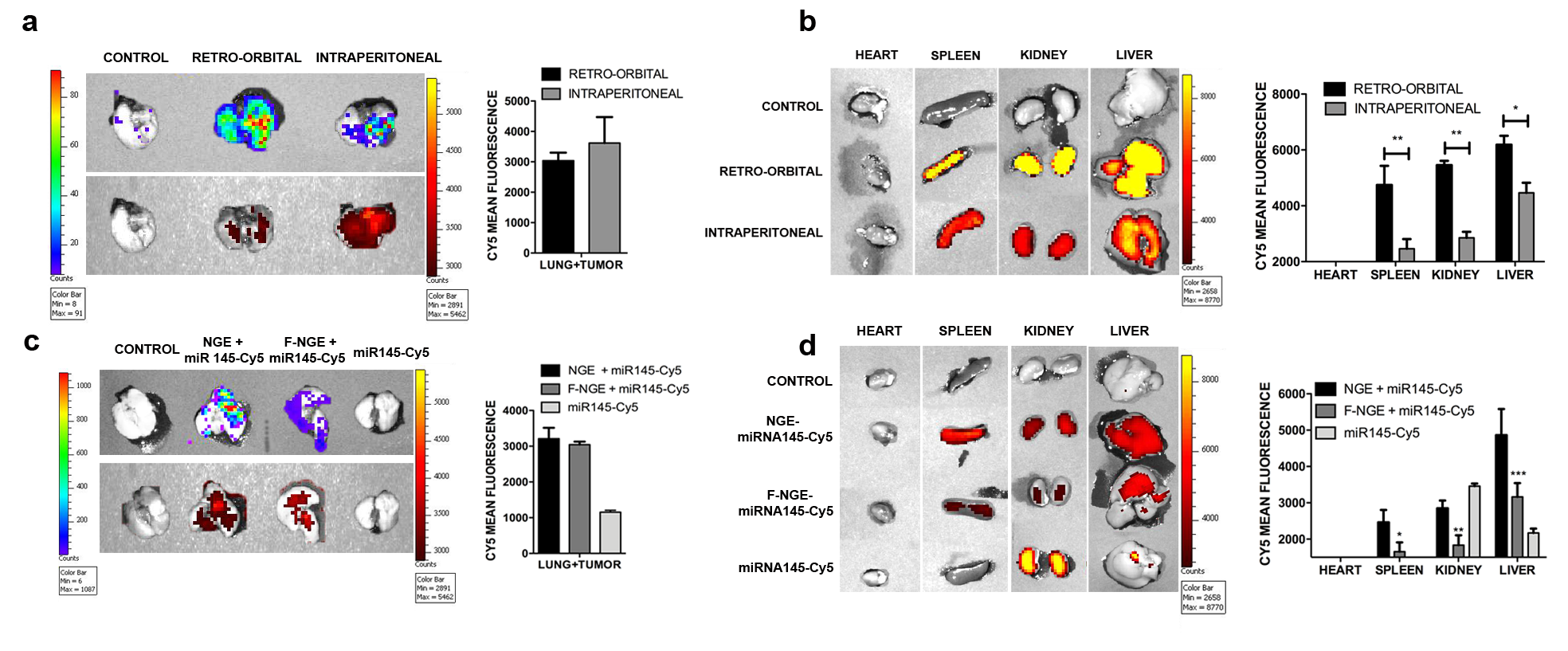
**

**a**

**b**

**Figure S9. Comparative *in vivo* biodistribution of two different administration routes (a-b)** Analysis of the retro-orbital and intraperitoneal inoculation methods of EMNs + miR145-Cy5. A representative image *ex vivo* (left) and quantification of the Cy5 fluorescence (right) of the **(a)** lung (tumor) and **(b)** indicated organs *ex vivo.* The scale bars (**a-c**) represent the luciferase intensity (left) and Cy5-fluorescence (right, arbitrary units). The data in graphs denote the mean values ± SEM from n=3 mice per condition and Cy5 fluorescence signal was normalized to the background obtained from tumors of mice control. *p<0.05; **0.001<p<0.05; ***p<0.001. Data without statistically significance were not indicated.
